# DNA methylation of IFI44L as a potential blood biomarker for childhood-onset systemic lupus erythematosus

**DOI:** 10.1038/s41390-024-03135-1

**Published:** 2024-03-21

**Authors:** Jingwei Wang, Xiqiang Dang, Xiaochuan Wu, Zhongyuan Xiang, Yongzhen Li, Yaqian Fu, Tian Shen

**Affiliations:** 1 Children’s Medical Center, The Second Xiangya Hospital, Central South University, Changsha, Hunan China; 2 Department of Pediatrics, The Second Xiangya Hospital, Central South University, Changsha, Hunan China; 3 Department of Laboratory Medicine, The Second Xiangya Hospital, Central South University, Changsha, Hunan China; 4 Health Management Center, The Second Xiangya Hospital of Central South University, Changsha, Hunan China

## Abstract

**Background:**

IFN-induced protein 44-like (*IFI44L*) promoter methylation has been demonstrated to serve as an effective blood diagnostic biomarker for adult-onset SLE. However, its utility as a diagnostic marker for childhood-onset SLE (cSLE) remains to be verified.

**Methods:**

Initially, we conducted a differential analysis of gene methylation and mRNA expression patterns in cSLE whole blood samples obtained from the public GEO database to determine *IFI44L* gene expression and assess the methylation status at its CpG sites. Subsequently, we collected clinical whole blood samples from 49 cSLE patients and 12 healthy children, employing an HRM-qPCR-based *IFI44L* methylation detection technique to evaluate its diagnostic efficacy in pediatric clinical practice.

**Results:**

A total of 26 hypomethylated, highly expressed genes in cSLE were identified by intersecting differentially expressed genes (DEGs) and differentially methylation genes (DMGs). GO enrichment analysis for these 26 genes indicated a robust association with type I IFN. Among the overlapping genes, *IFI44L* exhibited the most pronounced differential expression and methylation. In subsequent clinical validation experiments, *IFI44L* methylation was confirmed as an effective blood-based diagnostic biomarker for cSLE, achieving an AUC of 0.867, a sensitivity of 0.753, and a specificity of 1.000.

**Conclusions:**

*IFI44L* methylation is a promising blood biomarker for cSLE.

**Impact:**

- *IFI44L* promoter methylation was reported to serve as a highly sensitive and specific diagnostic marker for adult-onset SLE. However, the diagnostic efficacy of *IFI44L* in childhood-onset SLE (cSLE) still remains to be confirmed. In this study, we utilized bioinformatics analysis and conducted clinical experiments to demonstrate that *IFI44L* methylation can also serve as a promising blood biomarker for cSLE. The findings of this study can facilitate the diagnosis of cSLE and broaden our understanding of its molecular mechanisms, with a particular focus on those related to type I interferons.

## Introduction

Systemic lupus erythematosus (SLE) is a chronic, multi-system autoimmune disease characterized by autoantibody production, resulting in widespread inflammation and tissue damage throughout the body. The estimated prevalence of SLE ranges from 5 to 241 cases per 100,000 individuals, based on diverse population studies.^1^ Approximately only 15% of SLE patients experience disease onset during childhood or adolescence, leading to a relatively lower prevalence among children, estimated at around 33 to 88 cases per 100,000 people.^2,3^ In comparison to adult patients, those with childhood-onset systemic lupus erythematosus (cSLE) generally exhibit increased disease activity and more severe symptoms, affecting cardiopulmonary, renal and neuropsychiatric systems.^4^ However, diagnosing SLE in children is challenging due to its lower incidence rate in children and the symptom overlapped with other pediatric conditions.^2^ In clinical practice, the diagnosis of SLE always relies on autoantibodies such as antinuclear antibodies (ANA), anti-double-stranded DNA (dsDNA), and anti-Smith (SM) antibodies. However, these diagnostic markers all present limitations. Anti-ANA exhibits low specificity, while both anti-dsDNA and anti-Sm antibodies display inadequate sensitivity.^5–7^ Consequently, it is crucial to identify and validate novel efficient diagnostic biomarkers for cSLE.

Type I interferons (IFNs) were first observed to exhibit elevated levels in the serum of SLE patients during the 1980s, and subsequent research highlighted their critical role in SLE disease progression.^8,9^ A positive correlation has been reported between SLE disease activity and IFN-α levels, with an estimated 50–70% of patients exhibiting increased expression of type I interferon-stimulated genes (ISGs).^10–14^ One such ISG, interferon-induced protein 44-like (*IFI44L*), demonstrates notably enhanced expression in SLE cases.^15–18^ While the precise function of *IFI44L* in SLE is yet to be determined, multiple studies have shown a significant reduction in methylation levels within its gene promoter region among adult-onset SLE patients.^19–21^ Additionally, methylation at certain sites has been identified as a highly efficient biomarker for adult-onset SLE diagnosis.^22,23^ However, research on *IFI44L* in cSLE remains limited, and the potential value of DNA methylation in its promoter region as an effective diagnostic biomarker for cSLE warrants further investigation.

In this study, we performed a comprehensive analysis of differentially methylated sites and gene expression patterns between children with SLE and healthy controls to investigate the potential of *IFI44L* gene methylation as a diagnostic biomarker for cSLE. Subsequently, we employed a high-resolution melting quantitative PCR (HRM-qPCR)-based approach to detect *IFI44L* methylation in clinical samples, thereby validating its diagnostic utility in cSLE.

## Methods

### Public data acquisition

In this study, we obtained mRNA expression microarray (GSE65391) and genome-wide DNA methylation data (GSE118144) related to cSLE from the Gene Expression Omnibus (GEO, https://www.ncbi.nlm.nih.gov/geo/) database in the National Center for Biotechnology Information (NCBI). We incorporated whole blood samples collected at the initial onset and ultimately obtained a total of 138 cSLE samples and 52 healthy control (HC) samples from GSE65391, as well as 16 cSLE samples and 13 healthy control samples from GSE118144 (Supplementary File 1).

### Public data process

The mRNA expression data in GSE65391 was normalized, and differentially expressed genes (DEGs) were identified using the R package “Limma” by comparing the SLE group and the healthy control (HC) group. The cut-off criteria for DEGs were set at an adjust *p* < 0.05 and an absolute log_2_ fold change (|log_2_FC | ) ≥1. The DNA methylation data in GSE118144 was filtered using the “champ.filter” function in the R package “ChAMP”. Following this, the “champ.DMP” function was applied to identify differentially methylation probes (DMPs) with a threshold of an adjust *p* < 0.05 and an absolute difference in β levels (∆β) ≥0.1. DMPs mapped near the gene loci were assigned to the corresponding genes, which were identified as differentially methylation genes (DMGs). The probe exhibiting the greatest difference was chosen to represent the gene in cases where multiple corresponding methylation probes were present. Subsequently, DEGs and DMGs were overlapped to obtained 26 hypomethylated, highly expressed genes in cSLE (Supplementary Table S1). The corresponding DMPs of these overlapped genes can be found in Supplementary Table S2. And, we selected the top 15 with the significant differences in β levels for presentation in Table 1. The mRNA expression profile of the 26 hypomethylated, highly expressed genes was presented in Table 2. The detailed information regarding the corresponding DMPs of *IFI44L* was provided in Table 3. Adjusted *p*-value, along with log_2_FC or ∆β, were further processed to generate the volcano plot by using the R package “gplots”. The normalized expression data of DEGs or methylation levels of DMGs were employed to create the heatmap via the R package “pheatmap”. The R code used in this article can be found in Supplementary File 2.

**Table 1:** Childhood-onset SLE distinct DNA methylation signature.

Probe ID	Gene	Gene Group	Beta value of whole blood		
			Control	SLE	Difference
cg13452062	*IFI44L*	5′UTR	0.713	0.166	−0.547
cg21549285	*MX1*	5′UTR	0.735	0.309	−0.425
cg05696877	*IFI44L*	5′UTR	0.571	0.159	−0.412
cg12439472	*EPSTI1*	Body	0.614	0.269	−0.345
cg22930808	*PARP9*	5′UTR	0.661	0.330	−0.331
cg07815522	*PARP9*	5′UTR	0.672	0.358	−0.314
cg00959259	*PARP9*	5′UTR	0.539	0.243	−0.297
cg22862003	*MX1*	TSS1500	0.693	0.420	−0.273
cg08122652	*PARP9*	5′UTR	0.754	0.496	−0.259
cg01079652	*IFI44*	Body	0.897	0.642	−0.255
cg05552874	*IFIT1*	Body	0.626	0.372	−0.254
cg24678928	*DDX60*	TSS1500	0.786	0.535	−0.251
cg23570810	*IFITM1*	Body	0.708	0.468	−0.240
cg03607951	*IFI44L*	TSS1500	0.489	0.253	−0.236
cg01028142	*CMPK2*	Body	0.825	0.613	−0.212

This table presents detailed information of the top 15 corresponding DMPs associated with these hypomethylated, highly expressed genes with the significant differences in β levels in cSLE.

**Table 2:** Childhood-onset SLE distinct mRNA expression signature.

Gene	mRNA expression of whole blood		
	HC	SLE	log_2_FC
*IFI44L*	393.843	4845.204	4.032
*RSAD2*	217.026	2238.146	3.916
*IFI44*	523.023	3676.303	2.93
*ISG15*	986.898	6403.622	2.862
*IFIT3*	335.800	2133.825	2.773
*IFIT1*	141.922	938.985	2.763
*HERC5*	736.220	4186.448	2.678
*EPSTI1*	614.408	3373.188	2.626
*CMPK2*	17.416	104.671	2.487
*LY6E*	970.134	5029.074	2.339
*USP18*	18.634	112.943	2.311
*MX1*	1880.578	8370.341	2.200
*PLSCR1*	59.636	264.261	2.144
*DHX58*	87.350	349.268	2.005
*IFIT5*	74.106	282.966	1.897
*DDX60*	126.238	486.555	1.857
*LGALS3BP*	44.160	164.974	1.776
*PRIC285*	576.247	1803.682	1.668
*IFIH1*	249.174	794.372	1.62
*PARP9*	334.414	1045.296	1.612
*DDX58*	87.434	259.063	1.566
*PARP12*	302.447	920.821	1.494
*PARP14*	486.234	1279.183	1.309
*IRF7*	109.300	264.600	1.221
*IFITM1*	3562.358	8238.550	1.154
*SP100*	97.324	196.314	1.011

This table presents the gene expression profiles of these 26 hypomethylated, highly expressed genes in cSLE.

**Table 3:** Detailed information regarding the corresponding DMPs of *IFI44L*.

Probe ID	Gene	Gene Group	Map Information	Beta value of whole blood		
				HC	SLE	Difference
cg13452062	*IFI44L*	5′UTR	Chr1:79088559	0.713	0.166	−0.547
cg05696877	*IFI44L*	5′UTR	Chr1:79088769	0.571	0.159	−0.412
cg03607951	*IFI44L*	TSS1500	Chr1:79085586	0.489	0.253	−0.236
cg13304609	*IFI44L*	TSS1500	Chr1:79085162	0.832	0.684	−0.148
cg17980508	*IFI44L*	TSS1500	Chr1:79085713	0.336	0.207	−0.130

### Functional enrichment analysis

Gene ontology (GO) analysis were conducted on the hypomethylated, highly expressed genes by using the online tool Metascape (https://metascape.org) to gain a better understanding of their biological functions with the enrichment cutoff of Min overlaps ≥3 and *p* ≤ 0.05.

### Clinical information of patients and healthy controls

The study included 12 healthy children and 49 children with SLE, who were recruited from the Department of Pediatrics and the Pediatric Health Care Center at the Second Xiangya Hospital from January 1, 2022 to December 31, 2022. Clinical features including age, sex, systemic lupus erythematosus disease activity index (SLEDAI) scores (assessed by SLEDAI-2000^24^), concentration of complement 3 and complement 4, and titrations of autoimmune antibodies including anti-nuclear antibodies (ANAs) and anti-double strand DNA (dsDNA) were obtained from medical records and were presented in Supplementary Table S3. The baseline characteristics and diagnostic criteria of all patients in this study were presented in Table 4.

**Table 4:** The baseline characteristics and diagnostic criteria of research participants.

Group	HC	SLE
Sample size	12	49
Age (y) (mean ± SD)	12.3 ± 3.5	12.1 ± 2.8
Sex (%) Female	53.8	77.6
Diagnostic criteria		^31^

### Genomic DNA Isolation and Bisulfite

Genomic DNA was isolated from peripheral blood utilizing the GeneJET Whole Blood Genomic DNA Purification Mini Kit following the manufacturer’s instructions. The bisulfite conversion of the acquired genomic DNA was carried out using an EZ DNA Methylation™ Kit in accordance with the manufacturer’s guidelines. During Taq polymerase amplification, all unmethylated cytosines in the genome, except for 5-methyl-cytosines, were transformed into uracil and eventually into thymine.

### HRM-qPCR based *IFI44L* Methylation Detection System

In this study, we utilized a simple and highly efficient system, developed by Dr. Lu and validated in adult-onset SLE patients, to assess the diagnostic potential of *IFI44L* promoter methylation in our cSLE cohort.^23^ In this system, we initially cloned both the unmethylated (sequence A) and the fully methylated (sequence B) sequence within the *IFI44L* promoter region, containing two CG loci previously identified with methylation differences between SLE patients and healthy controls.^22^ Sequence A represented a fully unmethylated sequence (0% methylation) where the nucleotide CG is converted to TG, while sequence B represented a fully methylated sequence (100% methylation) retaining the CG nucleotide. We then separately inserted these two sequences into the pUC57 plasmid. Plasmids containing sequence A served as the 0% methylation standard (MS-0), and those containing sequence B served as the 100% methylation standard (MS-100). MS-0 and MS-100 was combined to create 25% (MS-25), 50% (MS-50), and 75% (MS-75) methylation standards by mixing at specific ratios of 3:1, 1:1, and 1:3. The melting curve of MS-25 served as a cut-off, categorizing samples with melting curves between MS-0 and MS-25 as positive, while those with melting curves between MS-25 and MS-100 were considered negative for SLE diagnosis.

The HRM-qPCR assay was executed on a LightCycler 96^®^ real-time PCR system (Roche). The reaction followed the manufacturer’s guidelines using the LightCycler^®^ 480 High Resolution Melting Master (Roche). Analysis of HRM curves was conducted with the LightCycler 96^®^ software (Roche), which normalized raw data according to fluorescence intensity, generating normalized HRM curves. The primer pair used for amplifying the target fragment of the *IFI44L* promoter were purchased from TSINGKE Company:

forward, 5′-GAAATGAAAGTAAGGAAGTTAGGAG-3′;

reverse, 5′-GGAATGGAGTGATAGTATTGGATTT-3′.

### Statistical analysis

Differences of continuous variables between two groups were assessed using independent *t*-test. Differences of categorical variables were examined using the Chi-square test. The area under the receiver operating characteristic (ROC) curve (AUC) was calculated. All analyses were performed using SPSS 26.0 or R 4.2.1software. *p* < 0.05 was considered statistically significant.

## Results

### Identification of differentially methylation genes (DMGs)

**Fig. 1:**
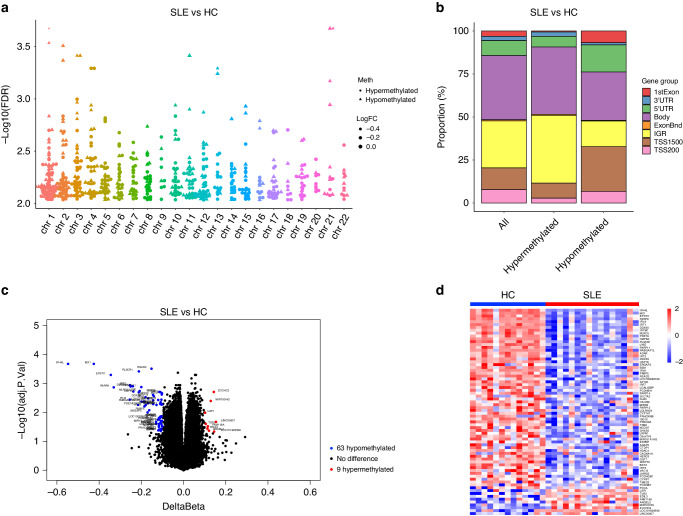
Identification of differentially methylation genes (DMGs). **a** A bar plot illustrating the distribution of DMPs’ locations across the entire genome. **b** A Manhattan plot displaying the epigenome-wide methylation results, with -log_10_(adjust *p*-value) comparison between SLE patients and healthy controls (HC). **c** A volcano plot showing DMGs between the SLE group and the HC group. Blue and red dots represent 63 hypomethylated and 9 hypermethylated genes in the SLE group. The significance thresholds setting as threshold of an adjust *p* < 0.05 and |∆β | ≥ 0.1. **d** A heat map showing DMGs between the SLE group and the HC group. Red color indicates hypermethylation while blue color indicates hypomethylation. Each row and column indicate one DMG and individual sample.

Regarding the DNA methylation data, 118 hypomethylated CpG sites within 63 genes and 16 hypermethylated CpG sites within 9 genes were identified in GSE118144 with a threshold of an adjust *p* < 0.05 and an absolute difference in β levels (|∆β | ) ≥0.1 (Fig. 1c, d). The differentially methylation CpG sites were not evenly distributed on autosomes (Fig. 1a). Additionally, in comparison to healthy children, cSLE patients exhibited enhanced demethylation near transcription start sites and the first exons, while demonstrating decreased demethylation in intergenic regions (IGRs) (Fig. 1b).

### Identification of differentially expressed genes (DEGs)

**Fig. 2:**
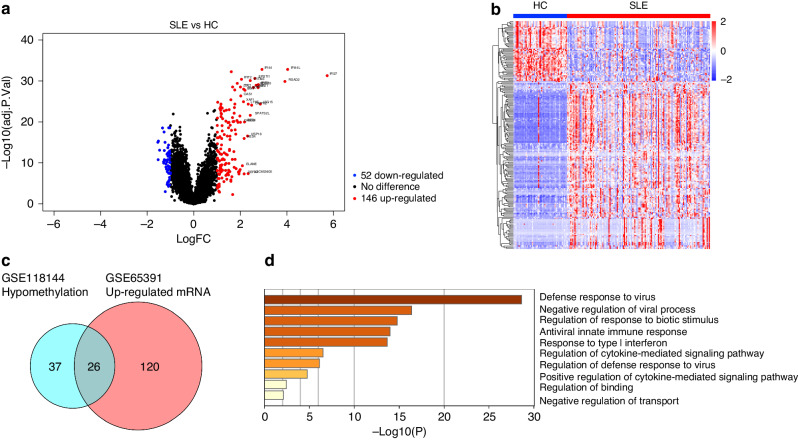
Identification of differentially expressed genes (DEGs) and gene enrichment analysis. **a** A volcano plot showing DEGs between the SLE group and HC group. Blue and red dots represent 52 down-regulated and 146 up-regulated genes in the SLE group. The significance thresholds setting as adjusted *p* < 0.05 and |log_2_FC | ≥ 1. **b** A heat map showing differentially expressed genes between the SLE group and the HC group. Red color indicates high gene expression while blue color indicates low gene expression. Each row and column indicate one DEG and individual sample. **c** A Venn diagram showing the hypomethylated, highly expressed genes in the SLE group. **d** A bar chart showing the significantly enriched terms associated with the hypomethylated, highly expressed genes in the SLE group.

As for mRNA expression data, whole blood samples of 138 cSLE patients and 52 healthy controls in GSE65391 were analyzed by using the R package “Limma” with the threshold of adjust *p* < 0.05 and |log_2_FC | ≥ 1. We identified a total of 198 DEGs in the SLE group compared to the HC group, consisting of 52 down-regulated genes and 146 up-regulated genes (Fig. 2a, b).

### The diagnostic performance of *IFI44L* methylation in cSLE

**Fig. 3:**
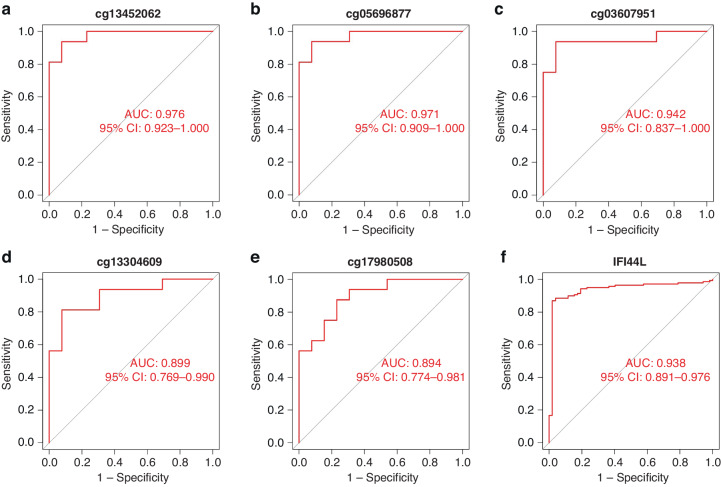
The diagnostic performance of *IFI44L* and its corresponding DMPs. **a**~**e** ROC curve analyses for the five corresponding DMPs of *IFI44LThe symbol f is present in Figure 3 but not mentioned in the legend. Please explain what this denotes.*. **b** ROC curve analysis of *IFI44L*.

A total of 26 genes were found to intersect between the hypomethylated genes in GSE118144 and the upregulated genes in GSE65391, which were identified as the hypomethylated, highly expressed genes (Fig. 2c). GO analysis conducted using the online website tool Metascape revealed that these 26 genes were significantly correlated with type I interferon signaling, such as defense response to virus and response to type I interferon (Fig. 2d). Within the top 15 differentially methylated positions (DMPs) associated with these genes, *IFI44L* occupied three prominent rankings, specifically the first, third, and fourteenth positions (Table 1). Moreover, the differential expression of *IFI44L* between cSLE patients and healthy children exhibits the greatest significance among the 26 overlapped genes (Table 2). A total of 5 CpG sites near the *IFI44L* loci displayed significant demethylation in cSLE patients. These included 3 sites (cg03607951, Chr1: 79085586; cg13304609, Chr1: 79085162; cg17980508, Chr1: 79085713) located within 1500 bp of the transcription start site (TSS, Chr1: 79086088) (Table 3). All 5 DMPs, in conjunction with *IFI44L* mRNA expression, demonstrated strong diagnostic performance in receiver operating characteristic (ROC) analyses, exhibiting area under the curve (AUC) values surpassing 0.7 (Fig. 3a~f).

### Validation of *IFI44L* methylation in clinical samples of cSLE using HRM-qPCR

**Fig. 4:**
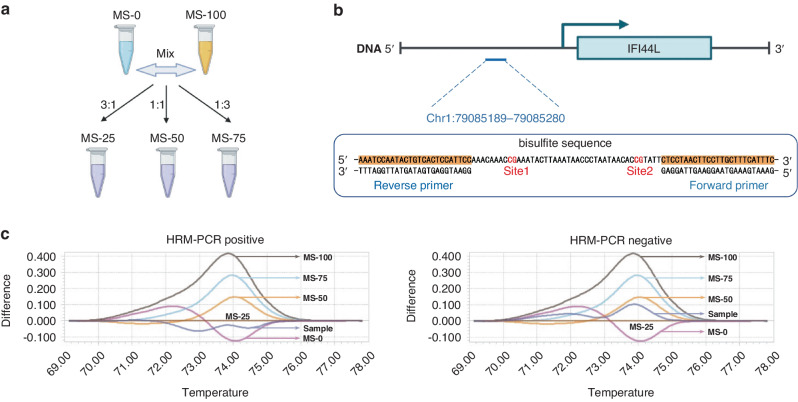
The HRM-qPCR based *IFI44L* methylation detection system. **a** Plasmids MS-0 and MS-100 were mixed to prepare MS-25, MS-50 and MS-75 in varying proportions. **b** The sequence of the loci adjacent to the *IFI44L* methylation site and the primers used for HRM-qPCR amplification. **c** Illustrative examples showcasing both positive and negative results within the system.

**Fig. 5:**
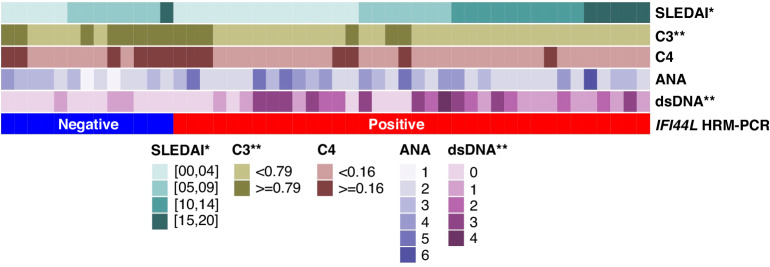
Clinical feature of SLE patients. A heatmap displaying the clinical data, comprising SLEDAI scores, complement 3 and complement 4 concentrations, as well as titrations of ANAs and anti-dsDNA for both HRM-qPCR positive and negative cSLE patients. Antibody titer: 0 ~negative, 1 ~ 1:40, 2 ~ 1:80, 3 ~ 1:160, 4 ~ 1:320, 5 ~ 1:640, 6 ~ 1:1280. *p < 0.05, **p < 0.01, ***p < 0.001.

An HRM-qPCR based simple and highly efficient method was applied in this study to validate the *IFI44L* methylation in our clinical samples. Plasmids containing sequence A (unmethylated sequence within the *IFI44L* promoter region, MS-0), and sequence B (fully methylated sequence within the *IFI44L* promoter region, MS-100) were mixed to create 25% (MS-25), 50% (MS-50), and 75% (MS-75) methylation standards by mixing at specific ratios of 3:1, 1:1, and 1:3 (Fig. 4a). The sequence of the loci near the methylation site and the primers used for HRM-qPCR amplification were shown in Fig. 4b. Samples with melting curves between MS-0 and MS-25 were considered HRM-qPCR positive, while those with melting curves between MS-25 and MS-100 were considered HRM-qPCR negative (Fig. 4c). Whole blood samples of 49 children with SLE and 12 healthy children were collected to detect the degree of *IFI44L* promoter methylation by HRM-qPCR. Among the 49 children diagnosed with SLE, 36 tested positive for *IFI44L* methylation, while all 12 healthy children tested negative (Supplementary File 3). This observation was statistically significant and confirmed by the chi-square test with the *p* < 0.05 (Table 5). The AUC was 0.867, the sensitivity was 0.753 and the specificity was 1.000 in the ROC analysis (Table 6). Figure 5 displayed clinical data for cSLE patients, encompassing SLEDAI scores, complement 3 (C3) and complement 4 (C4) concentrations, as well as titrations of ANAs and anti-dsDNA. Notably, significant differences were observed in SLEDAI scores, C3 concentrations, and anti-dsDNA titrations between the HRM-qPCR positive and negative cSLE patients. All data for Fig. 1 to Figure 5 and Tables 1 to 6 can be found in Supplementary File 4.

**Table 5:** Chi-square test for the 2 × 2 contingency table of *IFI44L* promoter region HRM-qPCR.

	HRM-qPCR			
Group	Positive	Negative	Total	*P*-value
HC	0	12	12	0.000
SLE	36	13	49	
Total	36	25	61

**Table 6:** The diagnostic value of HRM-qPCR in childhood-onset SLE.

AUC	Sensitivity	Specificity
0.867	0.735	1.000

This table presents the area under the curve (AUC), diagnostic sensitivity and diagnostic specificity from the receiver operating characteristic (ROC) curve analysis of *IFI44L* promoter region HRM-qPCR.

## Discussion

Type I interferons (IFNs) constitute a group of proteins crucial for immune system function, particularly in response to viral infections.^25–27^ In patients with systemic lupus erythematosus (SLE), earlier studies have demonstrated significant activation of the type I interferon system, an observation considered unrelated to antiviral activity but closely associated with SLE disease activity.^10^ With the approval of Anifrolumab—a novel therapeutic monoclonal antibody developed to target and inhibit the type I interferon receptor, consequently mitigating the downstream effects of type I IFNs—in August 2021 in the United States, the role of type I interferons in SLE has recaptured the interest of both the scientific community and the general public.^28^

Patients with SLE exhibit increased expression of type I IFN-stimulated genes, collectively referred to as the “IFN signature”. This signature is associated with more severe disease manifestations and poorer outcomes.^29^ A component of this signature, *IFI44L*, displays significant demethylation in its promoter region among SLE patients, leading to upregulated expression.^30^ Dr. Lu’s team identified two CpG methylation sites within the *IFI44L* promoter region in blood samples that could serve as biomarkers for adult-onset SLE diagnosis.^22^ They subsequently developed a simple and efficient method for detecting *IFI44L* methylation, utilizing HRM-qPCR targeting these specific sites. This technique proved nearly identical to pyrosequencing, the gold standard for gene methylation assessment, and exhibited a sensitivity of 88.571% and a specificity of 97.087% for adult-onset SLE diagnosis in clinical samples.^23^ Nevertheless, additional research is required to ascertain whether *IFI44L* methylation analysis can be extended to cSLE diagnosis.

In this study, to investigate the diagnostic potential of *IFI44L* methylation in cSLE. We acquired whole blood DNA methylation and mRNA expression data from two cSLE study cohorts in the GEO database. By conducting differential analyses of DNA methylation and mRNA expression between healthy children and those with SLE, we identified DMGs and DEGs in cSLE patients. Upon overlapping hypomethylated DMGs and upregulated DEGs, we pinpointed 26 hypomethylated, highly expressed genes in cSLE. GO enrichment analysis of these 26 genes demonstrated a strong association with type I IFN, consistent with findings in adult-onset SLE. Among these overlapped genes, *IFI44L* exhibited the most pronounced differential expression and methylation, with a ∆β of −0.547 and a log_2_ fold change (FC) of 4.03, highlighting its potential as a biomarker for cSLE diagnosis. ROC curve analyses confirmed that the all 5 DMPs of *IFI44L* offered exceptional diagnostic accuracy for cSLE.

To evaluate the clinical utility of *IFI44L* methylation, we collected whole blood samples from 49 children with SLE and 12 healthy children, implementing the HRM-qPCR-based *IFI44L* methylation detection method reported in Dr. Lu’s study.^23^ Among the cSLE patients, 36 out of 49 exhibited low methylation levels in the *IFI44L* promoter region, while all 12 healthy children displayed high methylation levels. ROC curve analysis revealed an AUC of 0.867, a sensitivity of 0.753, and a specificity of 1.000, indicating that *IFI44L* DNA methylation could serve as an efficient blood biomarker for cSLE diagnosis.

In comparison to previous adult study utilizing HRM-qPCR for *IFI44L* methylation detection, our clinical study demonstrated a specificity of 1.000, which is comparable to adult-onset SLE. However, its sensitivity was only 0.753, notably lower than the adult value of 0.886.^23^ We offered two potential explanations to address this concern. First, the adult *IFI44L* methylation sites (Chr1: 79085222 and Chr1: 79085250) may not necessarily represent the optimal sites for cSLE. Our findings suggested that Chr1:79085586, Chr1:79085162, and Chr1:79085713, situated within 1500 bp upstream of the *IFI44L* transcription start site, might serve as more appropriate choices for cSLE. Consequently, while HRM-qPCR remains viable for detecting *IFI44L* methylation in cSLE, it necessitates the design of distinct primers based on unique methylation sites compared to those used for adult-onset SLE. Second, by comparing the clinical data between HRM-qPCR negative and positive patients, we found significant differences in SLEDAI scores, C3 concentrations, and anti-dsDNA titrations. This suggests a potential relationship between HRM-qPCR results and SLE disease activity. In our clinical study, some of the clinical samples were obtained from children with inactive or mild active SLE (SLEDAI ≤ 9),^24^ which may lower the positive detection rate of HRM-qPCR. However, it is crucial to emphasize that the widely-used SLEDAI score for adult disease activity assessment may not be ideally suited for cSLE. For example, cSLE exhibits increased severity in cardiopulmonary and renal manifestations, whereas the SLEDAI scoring system inadequately evaluates cardiac and pulmonary lesions and offers only a generalized assessment of renal damage, failing to reflect kidney impairment severity at a pathological level. Hence, the ability of HRM-qPCR to accurately represent cSLE disease activity necessitates further exploration upon the development of a more precise and comprehensive cSLE activity evaluation system.

This study still had several limitations. First, we collected specimens from only 49 children with SLE, potentially introducing random errors into our results. Second, our study focused exclusively on comparing children with SLE to healthy children, overlooking a comprehensive and systematic investigation of various diseases that necessitate differentiation from cSLE in clinical pediatrics, including juvenile idiopathic arthritis, Kawasaki disease, dermatomyositis, polymyositis, Sjögren’s syndrome, systemic sclerosis, cytomegalovirus infection, and drug-induced lupus, etc. These limitations are scheduled to be addressed in our forthcoming clinical studies.

## Conclusions

In conclusion, our findings strongly indicate that *IFI44L* methylation a blood promising biomarker for cSLE patients. Nevertheless, the methylation sites necessary for clinical cSLE diagnosis could potentially vary from those observed in adult-onset SLE.

### Supplementary information

Supplementary 1

Supplementary 2

Supplementary 3

Supplementary 4

Supplementary 5

Supplementary Table S1

Supplementary Table S2

Supplementary Table S3

## Data Availability

The public datasets used and analyzed in this study are available from NCBI GEO: GSE65391. GSE118144.

## References

[1] Rees, F., Doherty, M., Grainge, M. J., Lanyon, P. & Zhang, W. The worldwide incidence and prevalence of systemic lupus erythematosus: a systematic review of epidemiological studies. *Rheumatology (Oxford)* **56**, 1945–1961 (2017). 28968809 10.1093/rheumatology/kex260

[2] Tucker, L. B. Making the diagnosis of systemic lupus erythematosus in children and adolescents. *Lupus* **16**, 546–549 (2007). 17711886 10.1177/0961203307078068

[3] Kamphuis, S. & Silverman, E. D. Prevalence and burden of pediatric-onset systemic lupus erythematosus. *Nat. Rev. Rheumatol.* **6**, 538–546 (2010). 20683438 10.1038/nrrheum.2010.121

[4] Joo, Y. B., Park, S. Y., Won, S. & Bae, S. C. Differences in clinical features and mortality between childhood-onset and adult-onset systemic lupus erythematosus: a prospective single-center study. *J. Rheumatol.* **43**, 1490–1497 (2016). 27252431 10.3899/jrheum.151129

[5] Nashi, R. A. & Shmerling, R. H. Antinuclear antibody testing for the diagnosis of systemic lupus erythematosus. *Rheum. Dis. Clin. North Am.* **48**, 569–578 (2022). 35400379 10.1016/j.rdc.2022.02.012

[6] Gensous, N. et al. Predictive biological markers of systemic lupus erythematosus flares: a systematic literature review. *Arthritis Res. Ther.* **19**, 238 (2017). 29065901 10.1186/s13075-017-1442-6 PMC5655881

[7] Shmerling, R. H. Diagnostic Tests for rheumatic disease: clinical utility revisited. *South Med. J.* **98**, 704–711 (2005). 16108239 10.1097/01.smj.0000171073.07875.c5

[8] Ytterberg, S. R. & Schnitzer, T. J. Serum interferon levels in patients with systemic lupus erythematosus. *Arthritis Rheum.* **25**, 401–406 (1982). 6176248 10.1002/art.1780250407

[9] Sibbitt, W. L. Jr., Froelich, C. J. & Bankhurst, A. D. Interferon-alpha regulation of lymphocyte function in systemic lupus erythematosus. *Clin. Immunol. Immunopathol.* **32**, 70–80 (1984). 6733982 10.1016/0090-1229(84)90044-8

[10] Bengtsson, A. A. et al. Activation of type I interferon system in systemic lupus erythematosus correlates with disease activity but not with antiretroviral antibodies. *Lupus* **9**, 664–671 (2000). 11199920 10.1191/096120300674499064

[11] Kirou, K. A. et al. Coordinate overexpression of interferon-alpha-induced genes in systemic lupus erythematosus. *Arthritis Rheum.* **50**, 3958–3967 (2004). 15593221 10.1002/art.20798

[12] Baechler, E. C. et al. Interferon-inducible gene expression signature in peripheral blood cells of patients with severe lupus. *Proc. Natl. Acad. Sci. USA* **100**, 2610–2615 (2003). 12604793 10.1073/pnas.0337679100 PMC151388

[13] Crow, M. K., Kirou, K. A. & Wohlgemuth, J. Microarray analysis of interferon-regulated genes in Sle. *Autoimmunity* **36**, 481–490 (2003). 14984025 10.1080/08916930310001625952

[14] Han, G. M. et al. Analysis of gene expression profiles in human systemic lupus erythematosus using oligonucleotide microarray. *Genes Immun.* **4**, 177–186 (2003). 12700592 10.1038/sj.gene.6363966

[15] Zhao, X. et al. Identification of key biomarkers and immune infiltration in systemic lupus erythematosus by integrated bioinformatics analysis. *J. Transl. Med.* **19**, 35 (2021). 33468161 10.1186/s12967-020-02698-x PMC7814551

[16] Luo, S., Wu, R., Li, Q. & Zhang, G. Epigenetic regulation of Ifi44l expression in monocytes affects the functions of monocyte-derived dendritic cells in systemic lupus erythematosus. *J. Immunol. Res.* **2022**, 4053038 (2022). 35592687 10.1155/2022/4053038 PMC9113863

[17] Wang, Y. et al. Identification of Ifi44l as a new candidate molecular marker for systemic lupus erythematosus. *Clin. Exp. Rheumatol.* **41**, 48–59 (2023). 35349411 10.55563/clinexprheumatol/q3aa6s

[18] Jiang, Z., Shao, M., Dai, X., Pan, Z. & Liu, D. Identification of diagnostic biomarkers in systemic lupus erythematosus based on bioinformatics analysis and machine learning. *Front Genet.* **13**, 865559 (2022). 35495164 10.3389/fgene.2022.865559 PMC9047905

[19] Salesi, M., Dehabadi, M. H., Salehi, R., Salehi, A. & Pakzad, B. Differentially methylation of Ifi44l gene promoter in Iranian patients with systemic lupus erythematosus and rheumatoid arthritis. *Mol. Biol. Rep.* **49**, 3065–3072 (2022). 35059970 10.1007/s11033-022-07134-5

[20] Zhang, B., Zhou, T., Wu, H., Zhao, M. & Lu, Q. Difference of Ifi44l methylation and serum Ifn-A1 level among patients with discoid and systemic lupus erythematosus and healthy individuals. *J. Transl. Autoimmun.* **4**, 100092 (2021). 33748734 10.1016/j.jtauto.2021.100092 PMC7972957

[21] Karimifar, M. et al. Interferon-induced protein 44-like gene promoter is differentially methylated in peripheral blood mononuclear cells of systemic lupus erythematosus patients. *J. Res. Med. Sci.* **24**, 99 (2019). 31850088 10.4103/jrms.JRMS_83_19 PMC6906918

[22] Zhao, M. et al. Ifi44l promoter methylation as a blood biomarker for systemic lupus erythematosus. *Ann. Rheum. Dis.* **75**, 1998–2006 (2016). 26787370 10.1136/annrheumdis-2015-208410 PMC4955646

[23] Zhang, B. et al. A simple and highly efficient method of Ifi44l methylation detection for the diagnosis of systemic lupus erythematosus. *Clin. Immunol.* **221**, 108612 (2020). 33069854 10.1016/j.clim.2020.108612

[24] Gladman, D. D., Ibanez, D. & Urowitz, M. B. Systemic lupus erythematosus disease activity index 2000. *J. Rheumatol.* **29**, 288–291 (2002). 11838846

[25] Lazear, H. M., Schoggins, J. W. & Diamond, M. S. Shared and distinct functions of type I and type III interferons. *Immunity* **50**, 907–923 (2019). 30995506 10.1016/j.immuni.2019.03.025 PMC6839410

[26] McNab, F., Mayer-Barber, K., Sher, A., Wack, A. & O’Garra, A. Type I interferons in infectious disease. *Nat. Rev. Immunol.* **15**, 87–103 (2015). 25614319 10.1038/nri3787 PMC7162685

[27] Mesev, E. V., LeDesma, R. A. & Ploss, A. Decoding Type I. and III interferon signalling during viral infection. *Nat. Microbiol.* **4**, 914–924 (2019). 30936491 10.1038/s41564-019-0421-x PMC6554024

[28] Deeks, E. D. Anifrolumab: first approval. *Drugs* **81**, 1795–1802 (2021). 34554438 10.1007/s40265-021-01604-z

[29] Crow, M. K. Type I interferon in the pathogenesis of lupus. *J. Immunol.* **192**, 5459–5468 (2014). 24907379 10.4049/jimmunol.1002795 PMC4083591

[30] He, Z. et al. Comprehensive analysis of epigenetic modifications and immune-cell infiltration in tissues from patients with systemic lupus erythematosus. *Epigenomics* **14**, 81–100 (2022). 34913398 10.2217/epi-2021-0318

[31] Sag, E. et al. Performance of the new SLICC classification criteria in childhood systemic lupus erythematosus: a multicentre study. *Clin. Exp. Rheumatol*. **32**, 440–444 (2014). 24642380

