# Supplementary material for: DNA methylation of IFI44L as a potential blood biomarker for childhood-onset systemic lupus erythematosus
Source: Pediatr Res. 2024 Mar 21;96(2):494–501. doi: 10.1038/s41390-024-03135-1 (PMC11343705; doi:10.1038/s41390-024-03135-1)
Supplement: Supplementary file 1 — Supplementary 1 [file 41390_2024_3135_MOESM1_ESM.pdf]

https://www.jianguoyun.com/p/DfgNEdIQ08zbCxihio0FIAA
