# Supplementary material for: DNA methylation of IFI44L as a potential blood biomarker for childhood-onset systemic lupus erythematosus
Source: Pediatr Res. 2024 Mar 21;96(2):494–501. doi: 10.1038/s41390-024-03135-1 (PMC11343705; doi:10.1038/s41390-024-03135-1)
Supplement: Supplementary file 2 — Supplementary 2 [file 41390_2024_3135_MOESM2_ESM.pdf]

````
---
title: "R Notebook"
output: html_notebook
---


```{r}
library(ChAMP)
beta = data.table::fread(input = "CHAMP/whole bloodGSE118144_filter.tsv",sep = "\t",header = T,stringsAsFactors = F,check.names = F,na.strings="NA",data.table = F)
p_value = data.table::fread(input = "CHAMP/whole bloodGSE118144_p.tsv",sep = "\t",header = T,stringsAsFactors = F,check.names = F,na.strings="NA",data.table = F)

rownames(p_value) = p_value$TargetID
p_value = as.matrix(p_value[, -1])

beta = beta
rownames(beta) = beta$TargetID
beta = beta[,-1]
beta=impute::impute.knn(as.matrix(beta))
sum(is.na(beta))
beta=beta$data
# beta=beta+0.00001
p_value = p_value[rownames(beta), ]

phenotype = data.table::fread(input = "CHAMP/whole bloodGSE118144_phe.tsv",sep = "\t",header = T,stringsAsFactors = F,check.names = F,na.strings="NA",data.table = F)
pd = phenotype[, c("sample", "group")]
colnames(pd) = c("Sample_Name", "group_list")
myLoad=champ.filter(beta = beta ,pd = pd, arraytype = "EPIC", detP = p_value) #这一步已经自动完成了过滤
group_list <- pd$group_list
myDMP <- champ.DMP(beta = myLoad$beta, pheno=group_list, arraytype = "EPIC", adjPVal = 1)
df_DMP <- myDMP$Control_to_SLE
logFC_t <- log2(1.2)
P.Value_t <- 0.05
# df_DMP$regulate <- ifelse(df_DMP$P.Value < P.Value_t & abs(df_DMP$logFC) > logFC_t,
#                                        ifelse(df_DMP$logFC > logFC_t ,'Up','Down'),'Stable')
df_DMP$regulate <- ifelse(df_DMP$adj.P.Val < P.Value_t & abs(df_DMP$deltaBeta) > 0.05 & df_DMP$B>=0, ifelse(df_DMP$deltaBeta > 0.05 ,'hypermethylated','hypomethylated'),'NA')
df_DMP = df_DMP[df_DMP$gene!="",]
df_DMP = df_DMP[order(abs(df_DMP$deltaBeta), decreasing = T),]
df_DMP = df_DMP[!duplicated(df_DMP$gene), ]
````
````

```
df_DMP$gene = as.character(df_DMP$gene)
print(table(df_DMP$regulate))
degs_labels = as.character(df_DMP$gene[df_DMP$regulate!="NA"])

library(ggVolcano)
plot_data = df_DMP[,c(14, 1:6,9,21)]
plot_data$gene = as.character(plot_data$gene)
write.table(plot_data, file = "GSE118144_degs.tsv", quote = F, sep = "\t", row.names = F,
                    col.names = T)
select_labels = c(plot_data$gene[plot_data$regulate=="hypermethylated"][1:10],
                          plot_data$gene[plot_data$regulate=="hypomethylated"][1:10])

rownames(plot_data) = plot_data$gene
volcano = ggvolcano(plot_data,
                            x = "logFC",
                            y = "adj.P.Val",
                            label = "gene",
                            output = FALSE,
                            legend_position = 'DL',
                            add_line = F,
                            custom_label = select_labels,
                            fills = c("#e64b35", "steelblue","gray"),
                            colors = c( "#e64b35","steelblue","gray"),
                            legend_title = "diffmethylation")



retain_exp = myLoad$beta[rownames(df_DMP), ]

expr_final = merge(df_DMP[, 14, drop=FALSE], retain_exp, by="row.names")
rownames(expr_final)= expr_final$gene
expr_final = expr_final[,c(-1,-2)]
DEGs_E = as.matrix(expr_final[degs_labels,])
ano_row = dplyr::filter(df_DMP, regulate!="NA")[, c(14,21)] %>%
dplyr::rename("Expression"="regulate")
rownames(ano_row) = ano_row$gene
ano_row = ano_row[order(ano_row$Expression, decreasing = T),-1,drop=FALSE]

ano_col = dplyr::rename(pd, "Type"="group_list")
rownames(ano_col) = ano_col$Sample_Name
ano_col = ano_col[order(ano_col$Type),-1,drop=FALSE]

type = c('#00AFBB', '#FFB6C1')
names(type) = c("Control", "SLE")
```

~~~
anno_colors = list()
anno_colors[["Type"]] = type
anno_colors[["Expression"]] = c('hypermethylated'='#e64b35','hypomethylated'="steelblue")
matrix = DEGs_E[rownames(ano_row), rownames(ano_col)]

library(pheatmap)
deg_pheat = pheatmap(matrix,
         cluster_rows = FALSE,
         show_rownames = F,
         show_colnames =   F,
          scale='row',
          cluster_cols = F,
          fontsize_row = 12,
          fontsize_col = 10,
         annotation_col = ano_col,
         annotation_row = ano_row,
         annotation_colors = anno_colors,
         border=F,
          #color = colorRampPalette(c("navy", "white", "firebrick3"))(100),
         annotation_names_row = F, annotation_names_col = F,
          color = colorRampPalette(c("#43609D","white","#e64b35"))(100),#换颜色
         ) #修改横轴坐标名倾斜度
          # filename = 'cor.fpkm1.png',)


ggsave(filename = 'volcano.pdf', height = 3.90, width = 5.03, plot = volcano, dpi = 400,path = 'GSE118144',units = 'in')
ggsave(filename = 'volcano.png', height = 3.90, width = 5.03, plot = volcano, dpi = 400,path = 'GSE118144',units = 'in')

ggsave(filename = 'pheatmap.pdf', height = 7.86, width = 8.25, plot = deg_pheat, dpi = 400,path = 'GSE118144',units = 'in')
ggsave(filename = 'pheatmap.png', height = 7.86, width = 8.25, plot = deg_pheat, dpi = 400,path = 'GSE118144',units = 'in')

```



```{r enrichments}
degs_table = plot_data[plot_data$regulate!="NA",] %>% dplyr::rename("Gene"="gene")
library(clusterProfiler)
library(org.Hs.eg.db)
gene <- bitr(degs_table$Gene, fromType = 'SYMBOL',toType = 'ENTREZID',OrgDb = org.Hs.eg.db)
~~~

```
keytypes(org.Hs.eg.db)
GO<-enrichGO(gene$ENTREZID,#GO 富集分析
             OrgDb = org.Hs.eg.db,
             keyType = "ENTREZID",#设定读取的 gene ID 类型
             ont = "ALL",#(ont 为 ALL 因此包括 Biological Process,Cellular Component,Mollecular Function 三部分）
             pvalueCutoff = 0.05,#设定 p 值阈值
             qvalueCutoff = 0.05,#设定 q 值阈值
               readable = T, minGSSize = 3,
  maxGSSize = 1000)
KEGG <- enrichKEGG(gene$ENTREZID,#KEGG 富集分析
                   organism = 'hsa',
                   pvalueCutoff = 0.05,
                  qvalueCutoff = 0.05,
                   minGSSize = 3, maxGSSize = 1000)
write.table(GO, file = 'GO_enrich.tsv', sep = '\t', row.names = F, col.names = T, quote = F)
KEGG2<-setReadable(KEGG, OrgDb = org.Hs.eg.db, keyType="ENTREZID")

write.table(KEGG2, file = 'KEGG_enrich.tsv', sep = '\t', row.names = F, col.names = T, quote = F)
```

clusterProfiler polts

```{r enrichements chord}
library(ggplot2)
library(GOplot)
library(ggpubr)
#GOplotIn<-GO[1:10,c(2,3,7,9)]
GOplotIn = KEGG2[order(KEGG2[,6]),c(1, 2,6,8)]
GOplotIn$geneID <-str_replace_all(GOplotIn$geneID,'/',',')
names(GOplotIn)<-c('ID','Term','adj_pval','Genes')
GOplotIn$Category = rep('KEGG',nrow(GOplotIn))
genedata<-data.frame(ID=degs_table$Gene,logFC=degs_table$deltaBeta)
circ_GO<-GOplot::circle_dat(GOplotIn, genedata)
Cicle = GOCircle(circ_GO,lfc.col = c('#e64b35','steelblue'), zsc.col=c("#43609D","white","#e64b35"), label.size = 4, nsub = 8)

ggsave(filename = 'KEGG_enrich.pdf', height = 6.29, width = 9.42, plot = Cicle, dpi = 400,path = '.',units = 'in')
ggsave(filename = 'KEGG_enrich.svg', height = 6.29, width = 9.42, plot = Cicle, dpi = 400,path = '.',units = 'in')



GO_enrich = dotplot(GO, color="pvalue",showCategory=10, split =
```

```
'ONTOLOGY',font.size=12)+facet_grid(ONTOLOGY~.,
scale="free")+scale_colour_distiller(palette = 'RdBu', type = 'seq', )+
  scale_size_area()

ggsave(filename = 'GO_enrich.pdf', height = 9.71, width = 11.98, plot = GO_enrich, dpi = 400,path = '.',units = 'in')
ggsave(filename = 'GO_enrich.svg', height = 9.71, width = 11.98, plot = GO_enrich, dpi = 400,path = '.',units = 'in')

enrich =   ggarrange(GO_enrich, Cicle, labels = LETTERS[1:2])


ggsave(filename = 'enrichd.pdf', height = 9.91, width = 18.00, plot = enrich, dpi = 400,path = '.',units = 'in')
ggsave(filename = 'enrichd.svg', height = 9.71, width = 18.00, plot = enrich, dpi = 400,path = '.',units = 'in')
```

```
#甲基化火山图
install.packages("calibrate")
res <- read.table("GSE118144_degs.txt", header=TRUE)
with(res, plot(deltaBeta, -log10(adj.P.Val), pch=20, main="Volcano plot", xlim=c(-0.6,0.6),ylim
=c(-0.15,5)))
with(subset(res, adj.P.Val<.05 & deltaBeta>0.1), points(deltaBeta, -log10(adj.P.Val), pch=20,
col="red"))
with(subset(res, adj.P.Val<.05 & deltaBeta<(-0.1)), points(deltaBeta, -log10(adj.P.Val),
pch=20, col="blue"))
library(calibrate)
with(subset(res, adj.P.Val<.05 & abs(logFC)>0.1), textxy(logFC, -log10(adj.P.Val), labs=gene,
cex=.5))

#甲基化热图
Exp=read.table("heatmap.txt",header=TRUE,row.names=1,check.names = FALSE)
EXP=log2(Exp)
library(pheatmap)
bk <- c(seq(-2,-0.1,by=0.01),seq(0,2,by=0.01))
pheatmap(Exp,
         color = c(colorRampPalette(colors =
c("blue","white"))(length(bk)/2),colorRampPalette(colors = c("white","red"))(length(bk)/2)),
         legend_breaks=seq(-8,8,2),
         breaks=bk,
         cluster_col = FALSE,cluster_row =
FALSE,show_rownames=T,show_colnames=F,scale="row",border_color = FALSE)

#甲基化 ROC 曲线
library(pROC)
expFile="input.txt"
geneFile="LASSOgene.txt"

rt=read.table(expFile, header=T, sep="\t", check.names=F, row.names=1)
y=gsub("(.*)\\_(.*)", "\\2", colnames(rt))
y=ifelse(y=="con", 0, 1)
geneRT=read.table(geneFile, header=F, sep="\t", check.names=F)
for(x in as.vector(geneRT[,1])){
  roc1=roc(y, as.numeric(rt[x,]))
  ci1=ci.auc(roc1, method="bootstrap")
  ciVec=as.numeric(ci1)
  pdf(file=paste0("ROC.",x,".pdf"), width=5, height=5)
  plot(roc1, print.auc=TRUE, col="red", legacy.axes=T, main=x)
  text(0.39, 0.43, paste0("95% CI: ",sprintf("%.03f",ciVec[1]),"-",sprintf("%.03f",ciVec[3])),
col="red")
  dev.off()
```

```
}

#mRNA 差异分析
library('gplots')
library('limma')
rawexprSet=read.table("fpkm.txt",header=TRUE,row.names=1,check.names = FALSE)
dim(rawexprSet)
exprSet=log2(rawexprSet)
par(mfrow=c(1,2))
boxplot(data.frame(exprSet),col="blue")
dev.off()
exprSet[1:5,1:5]
group <- read.table("design.txt",header=TRUE,row.names=1,check.names = FALSE)
group <- group[,1]
design <- model.matrix(~0+factor(group))
colnames(design)=levels(factor(group))
rownames(design)=colnames(exprSet)
fit <- lmFit(exprSet,design)
cont.matrix<-makeContrasts(paste0(unique(group),collapse = "-"),levels = design)
fit2=contrasts.fit(fit,cont.matrix)
fit2 <- eBayes(fit2)
tempOutput = topTable(fit2,coef=1,n=Inf,adjust="BH")
nrDEG = na.omit(tempOutput)
allDiff <- nrDEG
diff=allDiff
write.table(diff, "limmaOut.txt",sep="\t",quote=F,col.names=T)

#mRNA 火山图
install.packages("calibrate")
res <- read.table("limmaOut.txt", header=TRUE)
with(res, plot(logFC, -log10(adj.P.Val), pch=20, main="Volcano plot", xlim=c(-1.5,1.5),ylim=c(-0.15,12)))
with(subset(res, adj.P.Val<.05 & logFC>0.5), points(logFC, -log10(adj.P.Val), pch=20, col="red"))
with(subset(res, adj.P.Val<.05 & logFC<(-0.5)), points(logFC, -log10(adj.P.Val), pch=20, col="blue"))
library(calibrate)
with(subset(res, adj.P.Val<.05 & abs(logFC)>1), textxy(logFC, -log10(adj.P.Val), labs=gene, cex=.5))

#mRNA 热图
Exp=read.table("heatmap.txt",header=TRUE,row.names=1,check.names = FALSE)
EXP=log2(Exp)
library(pheatmap)
```

```
bk <- c(seq(-2,-0.1,by=0.01),seq(0,2,by=0.01))
pheatmap(Exp,
         color = c(colorRampPalette(colors = c("blue","white"))(length(bk)/2),colorRampPalette(colors = c("white","red"))(length(bk)/2)),
         legend_breaks=seq(-8,8,2),
         breaks=bk,
         cluster_col = FALSE,cluster_row = TRUE,show_rownames=F,show_colnames=F,scale="row",border_color = FALSE)

#mRNAROC 曲线
library(pROC)
expFile="input.txt"
geneFile="LASSOgene.txt"
rt=read.table(expFile, header=T, sep="\t", check.names=F, row.names=1)
y=gsub("(.*)\\_(.*)", "\\2", colnames(rt))
y=ifelse(y=="con", 0, 1)
geneRT=read.table(geneFile, header=F, sep="\t", check.names=F)
for(x in as.vector(geneRT[,1])){
  #????ROC????
  roc1=roc(y, as.numeric(rt[x,]))
  ci1=ci.auc(roc1, method="bootstrap")
  ciVec=as.numeric(ci1)
  pdf(file=paste0("ROC.",x,".pdf"), width=5, height=5)
  plot(roc1, print.auc=TRUE, col="red", legacy.axes=T, main=x)
  text(0.39, 0.43, paste0("95% CI: ",sprintf("%.03f",ciVec[1]),"-",sprintf("%.03f",ciVec[3])), col="red")
  dev.off()
}

#临床信息热图
library(ComplexHeatmap)
riskFile="risk.txt"
cliFile="clinical1.txt"
risk=read.table(riskFile, header=T, sep="\t", check.names=F, row.names=1)
cli=read.table(cliFile,sep="\t",header=T,check.names=F,row.names=1)
samSample=intersect(row.names(risk), row.names(cli))
risk=risk[samSample,"Risk",drop=F]
cli=cli[samSample,,drop=F]
rt=cbind(cli, risk)
sigVec=c()
for(clinical in colnames(rt[,1:(ncol(rt)-1)])){
  data=rt[c("Risk", clinical)]
  colnames(data)=c("Risk", "clinical")
  data=data[(data[,"clinical"]!="unknow"),]
```

```
    tableStat=table(data)
    stat=chisq.test(tableStat)
    pvalue=stat$p.value
    Sig=ifelse(pvalue<0.001,"***",ifelse(pvalue<0.01,"**",ifelse(pvalue<0.05,"*","")))
    sigVec=c(sigVec, paste0(clinical, Sig))
    #print(tableStat)
    #print(paste(clinical, pvalue, Sig, sep="\t"))
}
sigVec=c(sigVec, "Risk")
colnames(rt)=sigVec
rt$Risk=factor(rt$Risk, levels=c("low","high"))
bioCol=c("#D1E9E9","#95CACA","#4F9D9D","#336666", "#C2C287", "#808040", "#CF9E9E", "#804040", "#F3F3FA",
         "#D8D8E8", "#B8B8DC", "#9999CC", "#7373B9", "#5151A2", "#EBD3E8", "#D2A2CC", "#B766AD", "#8F4586",
         "#6C3365")
colorList=list()
j=0
for(cli in colnames(rt[,1:(ncol(rt)-1)])){
    cliLength=length(levels(factor(rt[,cli])))
    cliCol=bioCol[(j+1):(j+cliLength)]
    j=j+cliLength
    names(cliCol)=levels(factor(rt[,cli]))
    cliCol["unknow"]="grey75"
    colorList[[cli]]=cliCol
}
colorList[["Risk"]]=c("low"="blue", "high"="red")
ha=HeatmapAnnotation(df=rt, col=colorList)
zero_row_mat=matrix(nrow=0, ncol=nrow(rt))
Hm=Heatmap(zero_row_mat, top_annotation=ha)
pdf(file="cliheatmap.pdf", width=7, height=5)
draw(Hm, merge_legend=TRUE, heatmap_legend_side="bottom", annotation_legend_side="bottom")
dev.off()
```
