# Supplementary material for: DNA methylation of IFI44L as a potential blood biomarker for childhood-onset systemic lupus erythematosus
Source: Pediatr Res. 2024 Mar 21;96(2):494–501. doi: 10.1038/s41390-024-03135-1 (PMC11343705; doi:10.1038/s41390-024-03135-1)
Supplement: Supplementary file 3 — Supplementary 3 [file 41390_2024_3135_MOESM3_ESM.pdf]

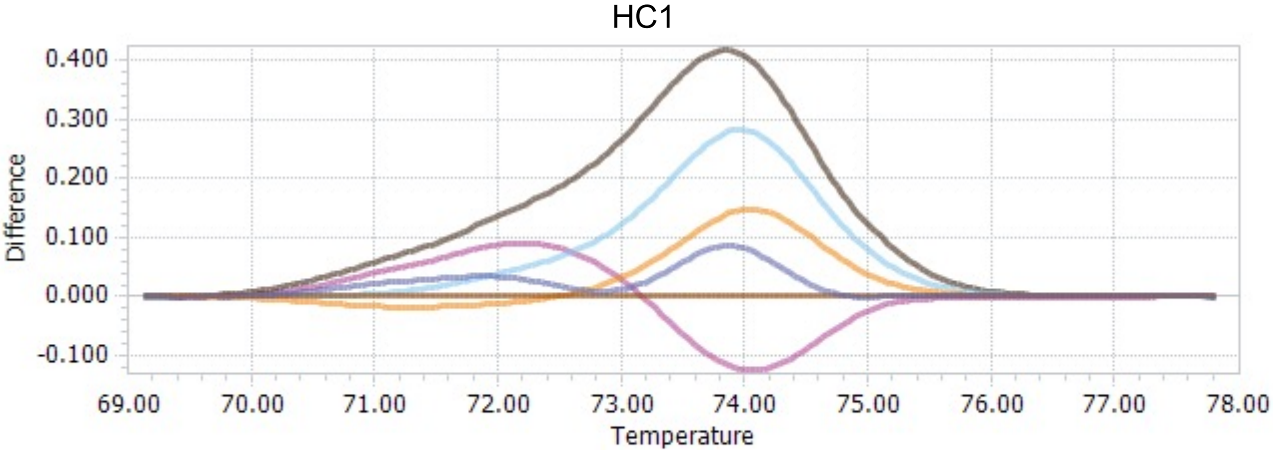
HC1
Difference
0.400
0.300
0.200
0.100
0.000
-0.100
69.00
70.00
71.00
72.00
73.00
74.00
75.00
76.00
77.00
78.00
Temperature

HC2

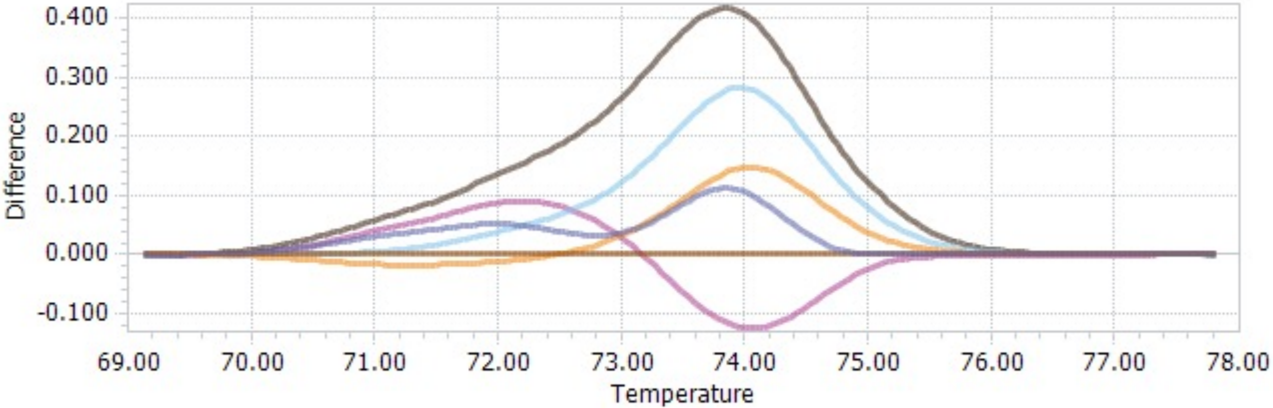

0.400
0.300
0.200
0.100
0.000
-0.100
Difference
69.00
70.00
71.00
72.00
73.00
74.00
75.00
76.00
77.00
78.00
Temperature

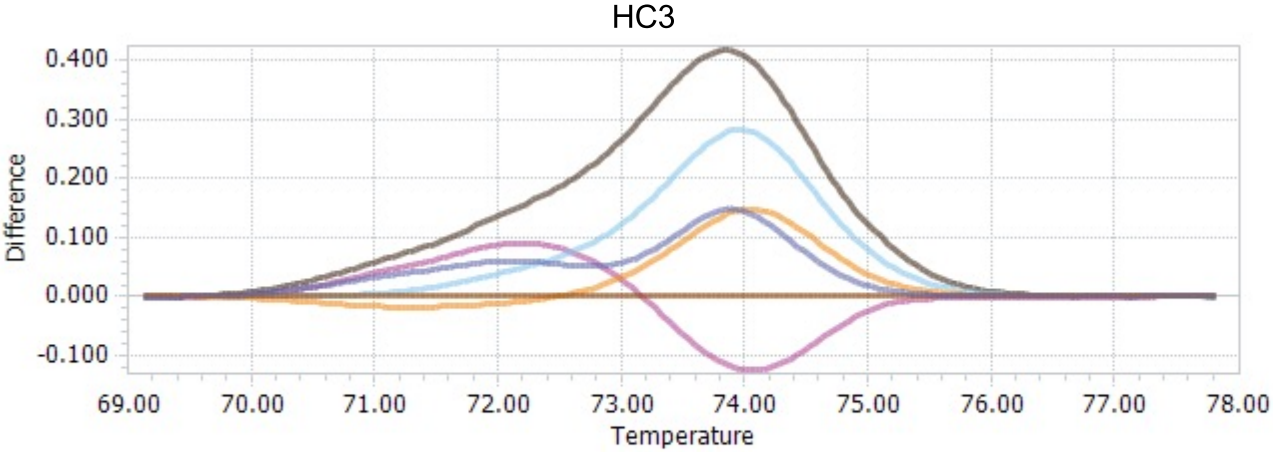
HC3
Difference
0.400
0.300
0.200
0.100
0.000
-0.100
69.00
70.00
71.00
72.00
73.00
74.00
75.00
76.00
77.00
78.00
Temperature

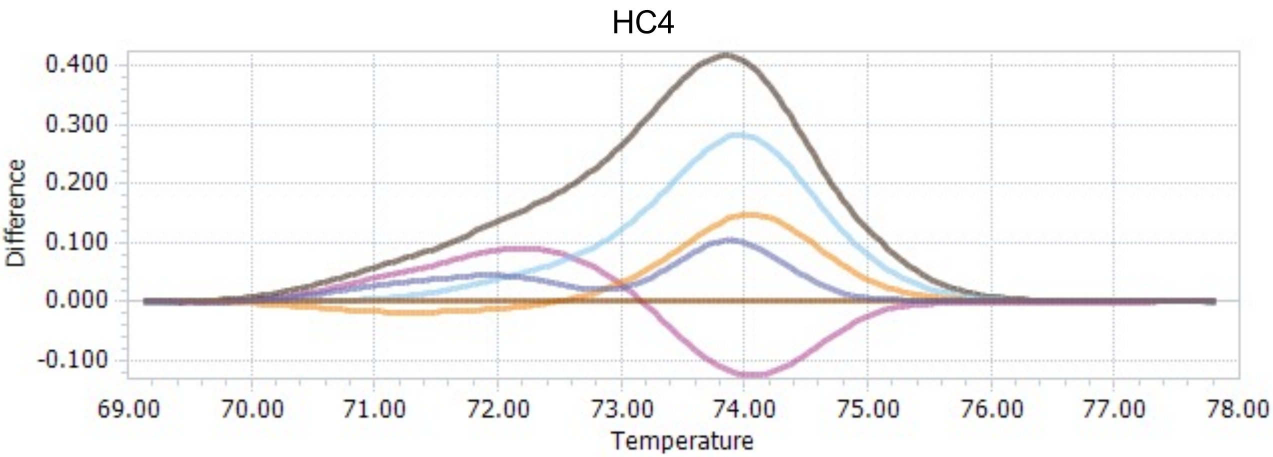
HC4
Difference
0.400
0.300
0.200
0.100
0.000
-0.100
69.00
70.00
71.00
72.00
73.00
74.00
75.00
76.00
77.00
78.00
Temperature

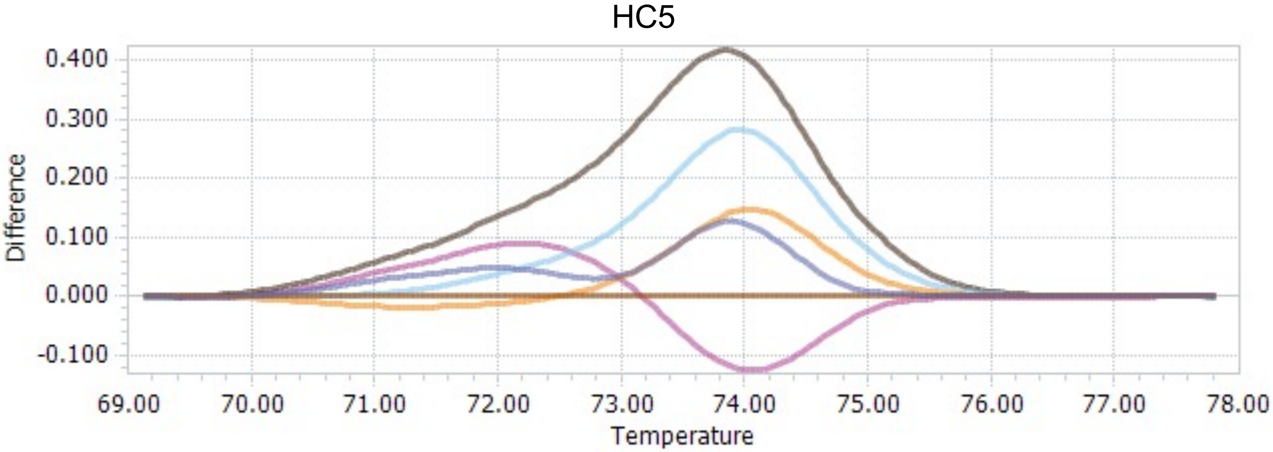

HC5
0.400
0.300
0.200
0.100
0.000
-0.100
Difference
69.00
70.00
71.00
72.00
73.00
74.00
75.00
76.00
77.00
78.00
Temperature

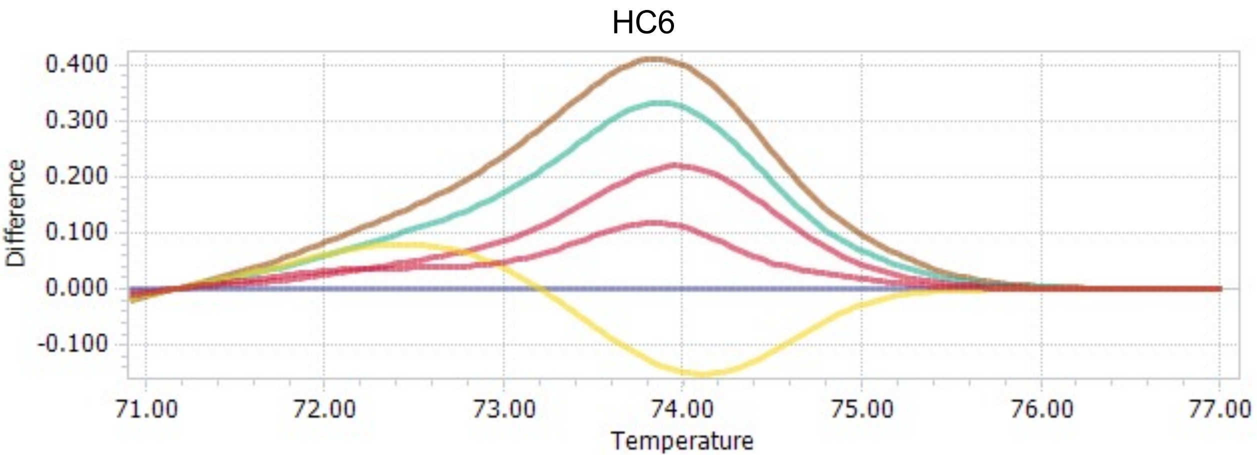
HC6
0.400
0.300
0.200
0.100
0.000
-0.100
Difference
71.00
72.00
73.00
74.00
75.00
76.00
77.00
Temperature

HC7

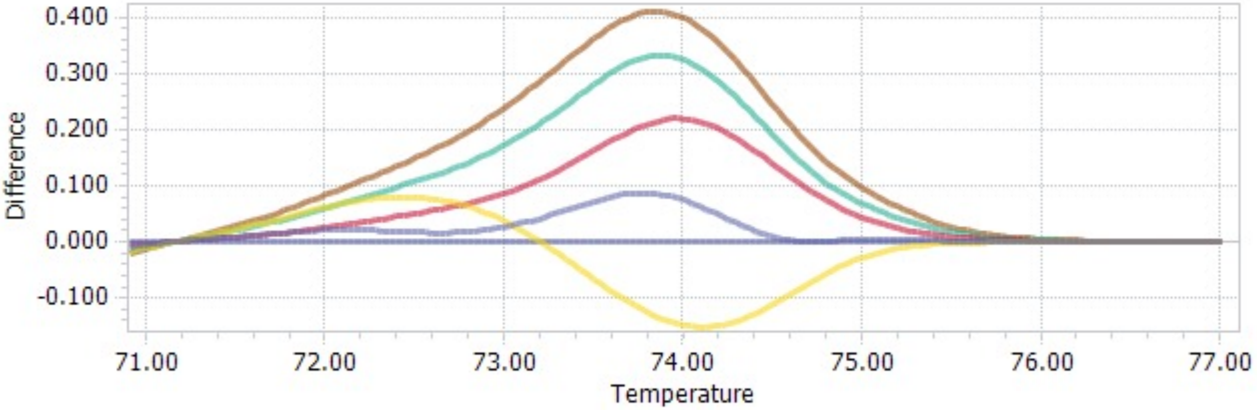
Difference
0.400
0.300
0.200
0.100
0.000
-0.100
71.00
72.00
73.00
74.00
75.00
76.00
77.00
Temperature

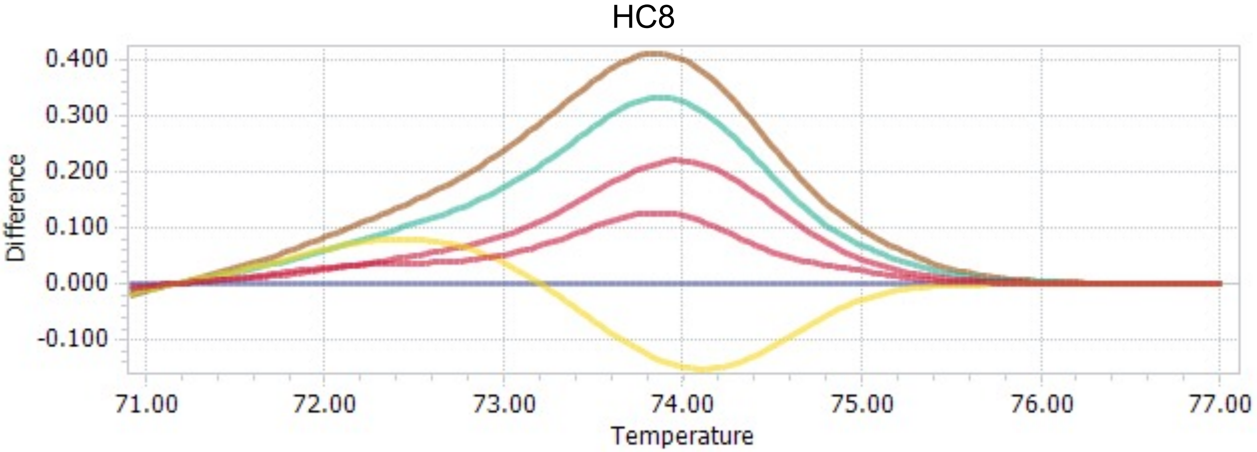
HC8
0.400
0.300
0.200
0.100
0.000
-0.100
Difference
71.00
72.00
73.00
74.00
75.00
76.00
77.00
Temperature

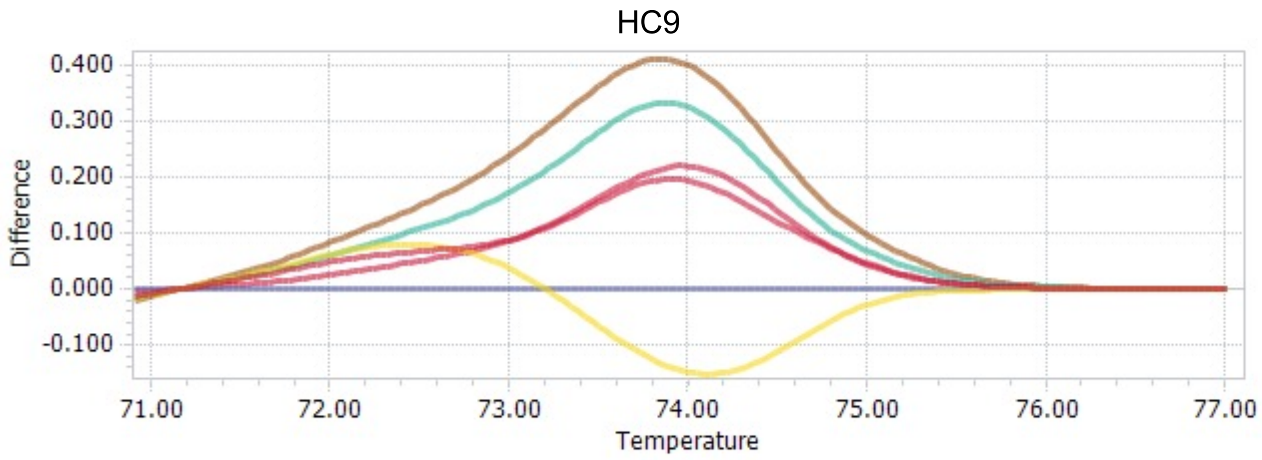
HC9
0.400
0.300
0.200
0.100
0.000
-0.100
Difference
71.00
72.00
73.00
74.00
75.00
76.00
77.00
Temperature

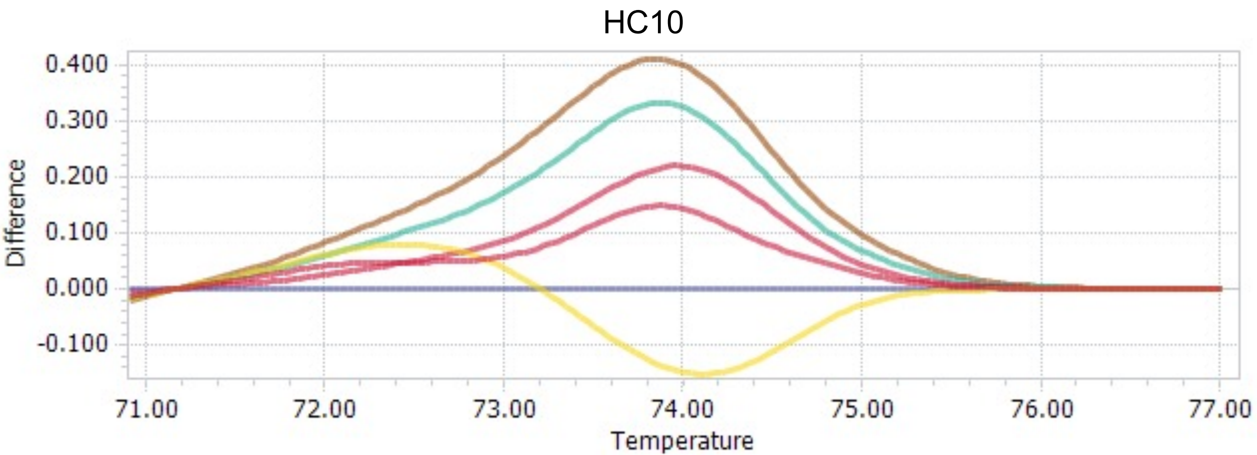
HC10
Difference
0.400
0.300
0.200
0.100
0.000
-0.100
71.00
72.00
73.00
74.00
75.00
76.00
77.00
Temperature

HC11

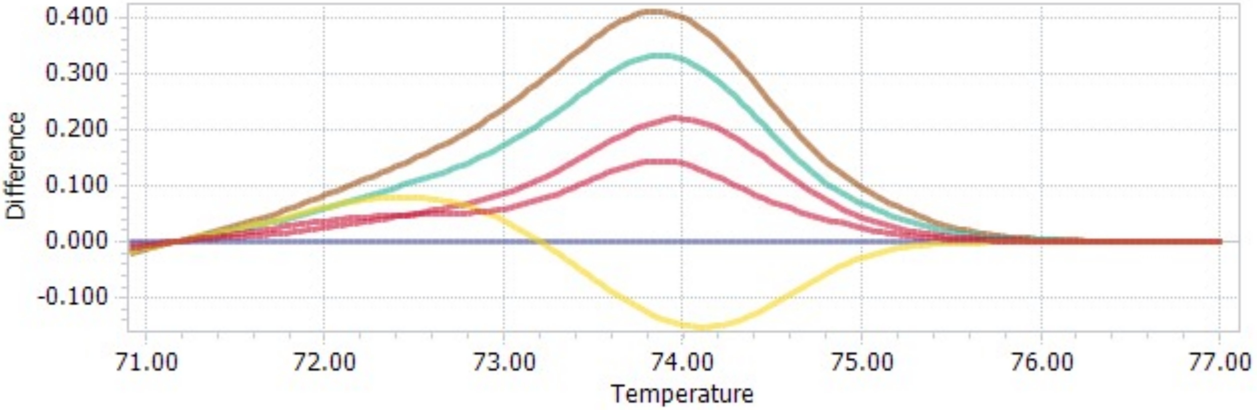

0.400
0.300
0.200
0.100
0.000
-0.100
Difference
71.00
72.00
73.00
74.00
75.00
76.00
77.00
Temperature

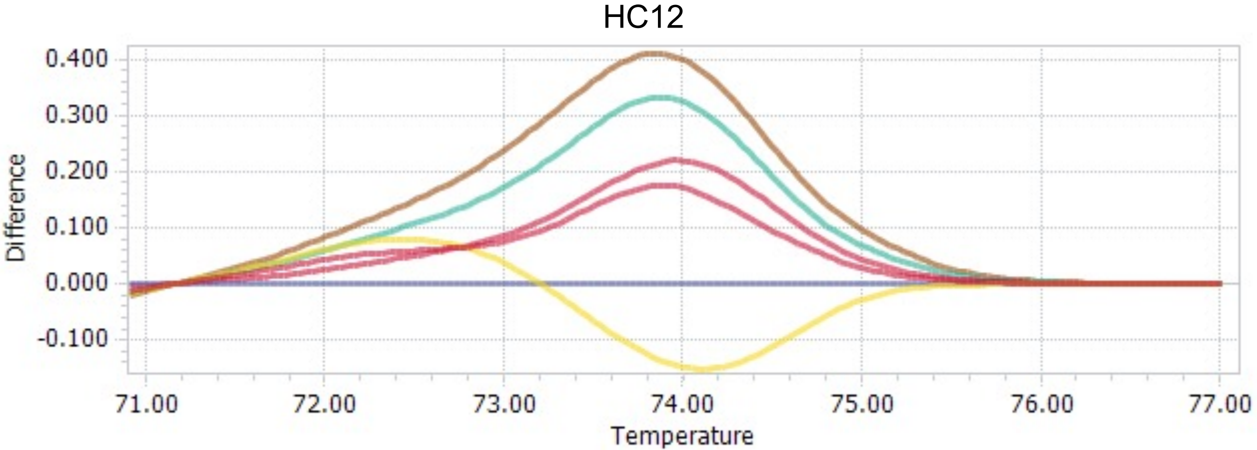
HC12
0.400
0.300
0.200
0.100
0.000
-0.100
Difference
71.00
72.00
73.00
74.00
75.00
76.00
77.00
Temperature

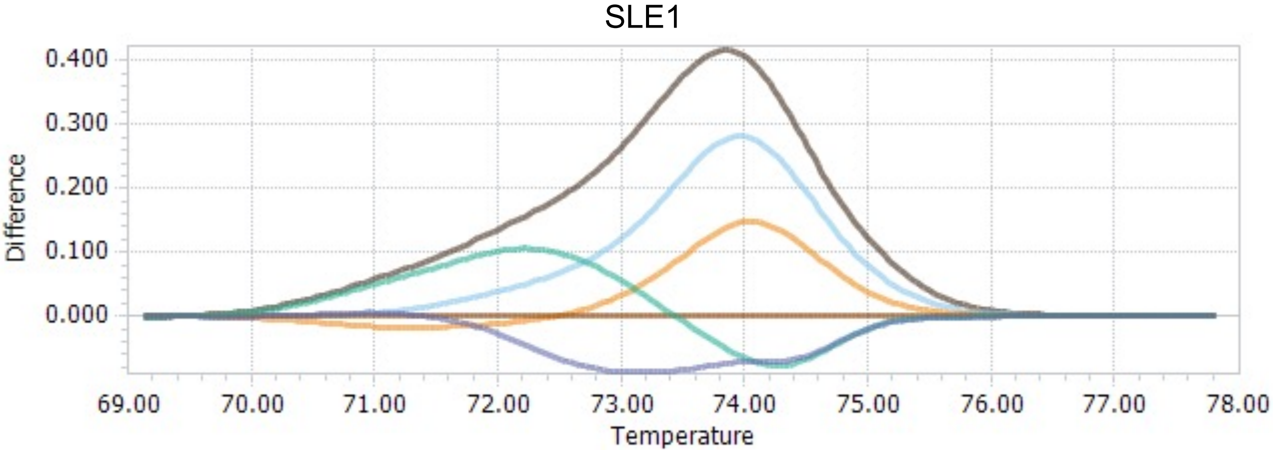
SLE1
0.400
0.300
0.200
0.100
0.000
Difference
69.00
70.00
71.00
72.00
73.00
74.00
75.00
76.00
77.00
78.00
Temperature

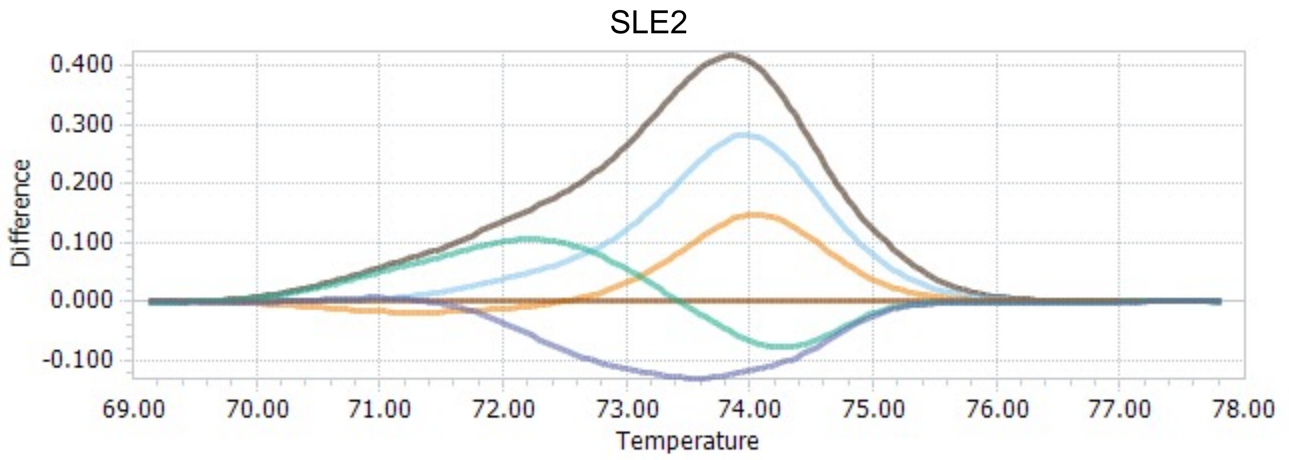
SLE2
0.400
0.300
0.200
0.100
0.000
-0.100
Difference
69.00
70.00
71.00
72.00
73.00
74.00
75.00
76.00
77.00
78.00
Temperature

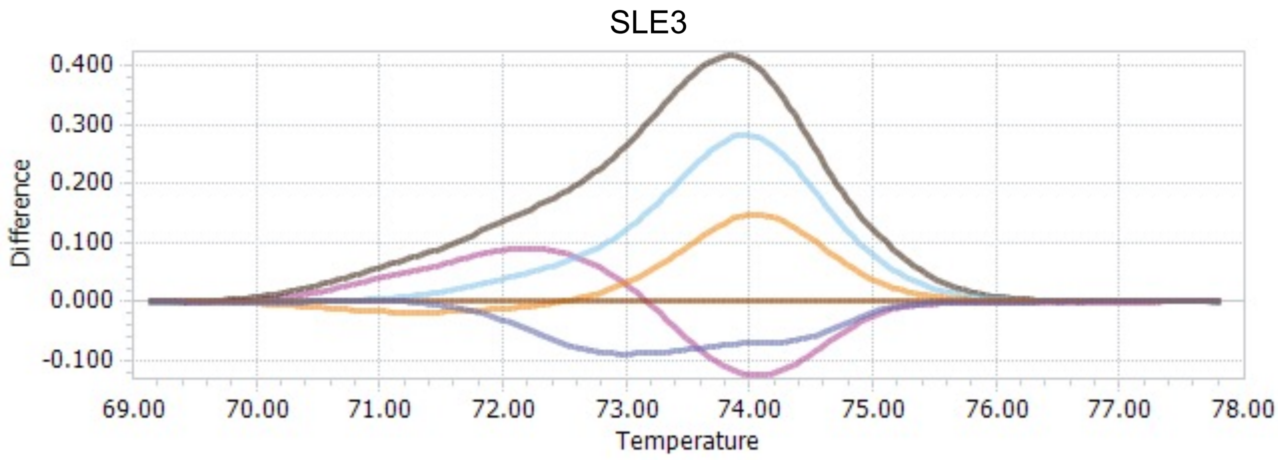
SLE3
Difference
0.400
0.300
0.200
0.100
0.000
-0.100
69.00
70.00
71.00
72.00
73.00
74.00
75.00
76.00
77.00
78.00
Temperature

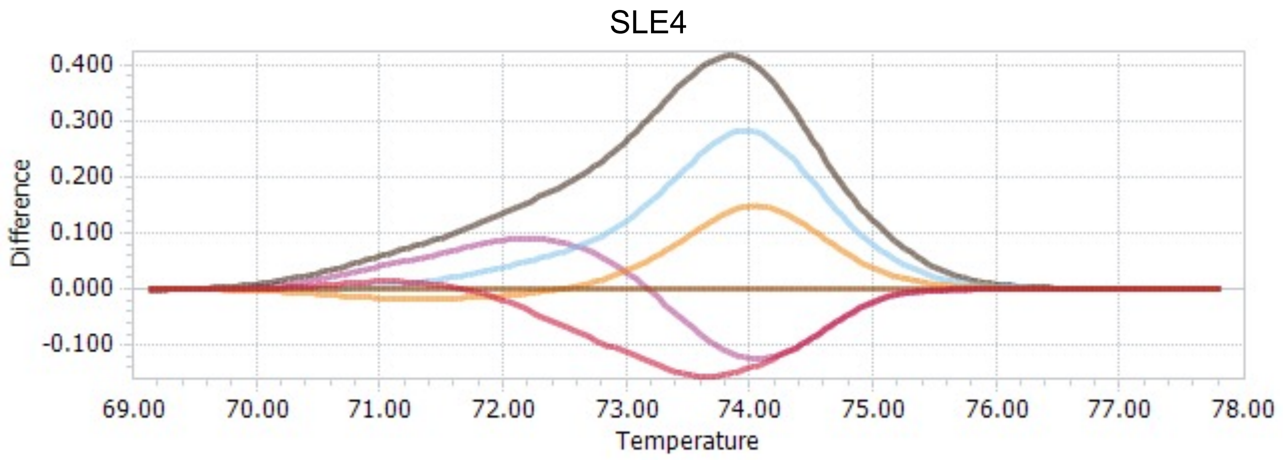
SLE4
Difference
0.400
0.300
0.200
0.100
0.000
-0.100
69.00
70.00
71.00
72.00
73.00
74.00
75.00
76.00
77.00
78.00
Temperature

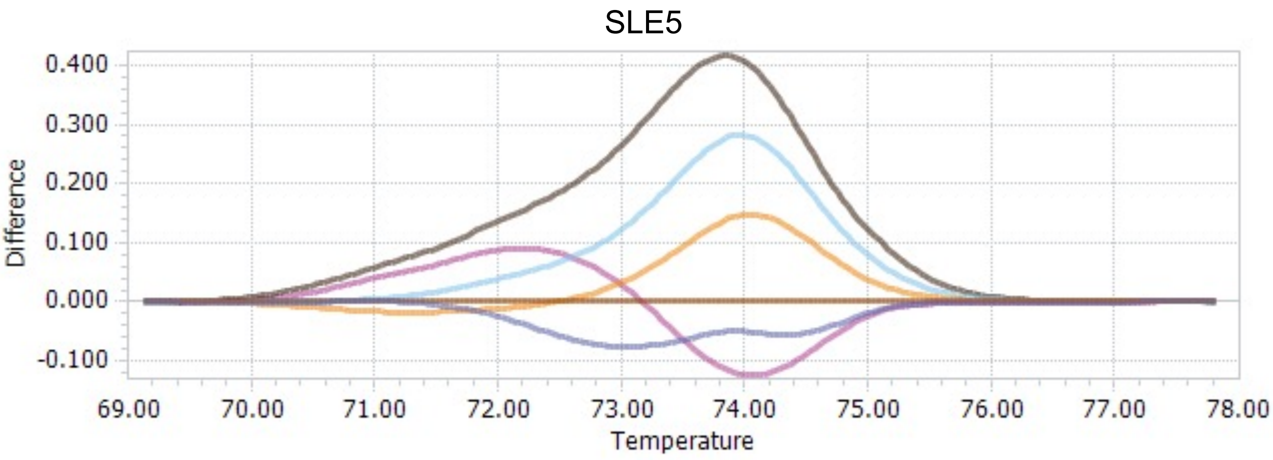
SLE5
Difference
0.400
0.300
0.200
0.100
0.000
-0.100
69.00
70.00
71.00
72.00
73.00
74.00
75.00
76.00
77.00
78.00
Temperature

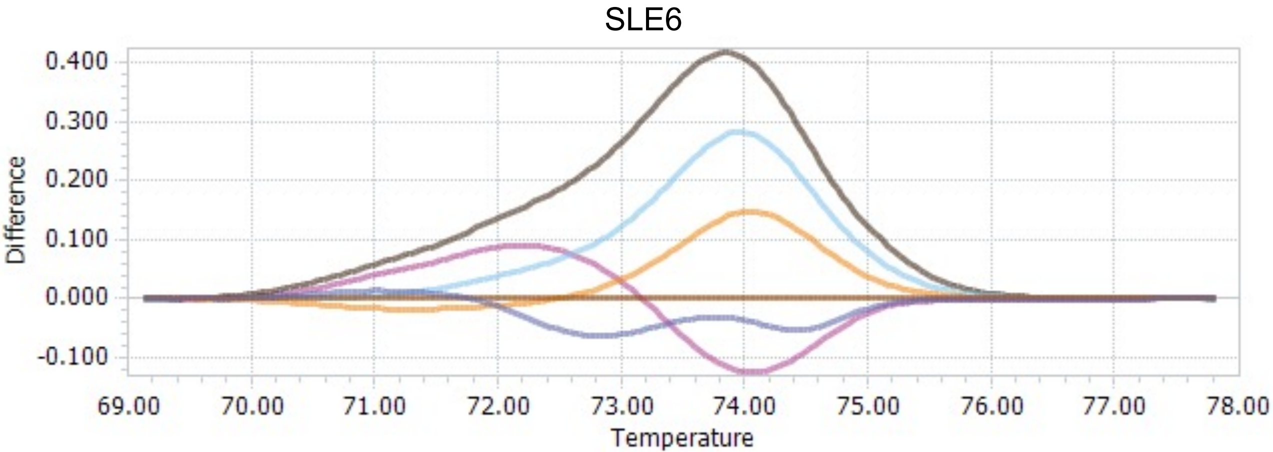

SLE6
Difference
0.400
0.300
0.200
0.100
0.000
-0.100
69.00
70.00
71.00
72.00
73.00
74.00
75.00
76.00
77.00
78.00
Temperature

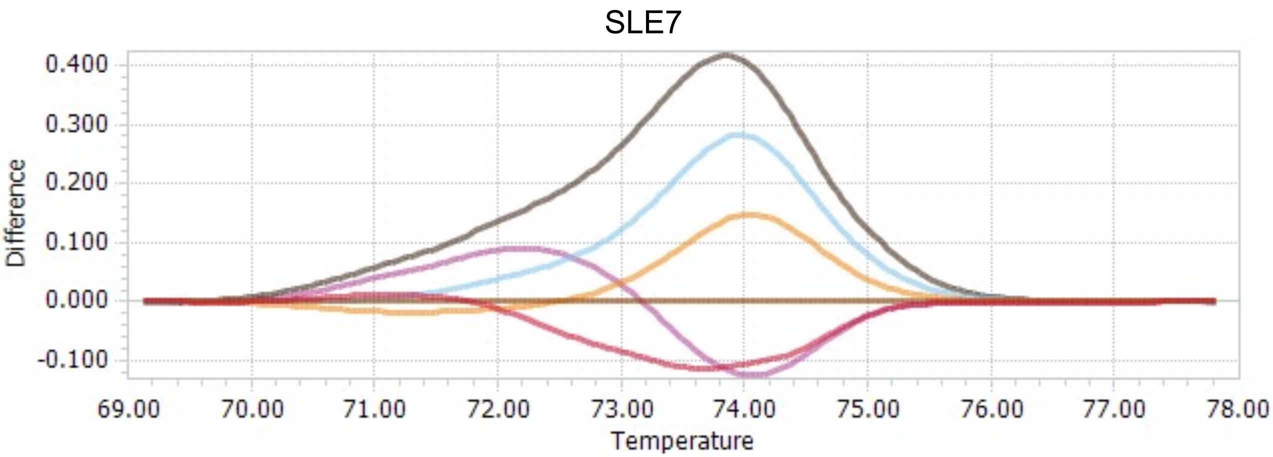
SLE7
0.400
0.300
0.200
0.100
0.000
-0.100
Difference
69.00
70.00
71.00
72.00
73.00
74.00
75.00
76.00
77.00
78.00
Temperature

SLE8

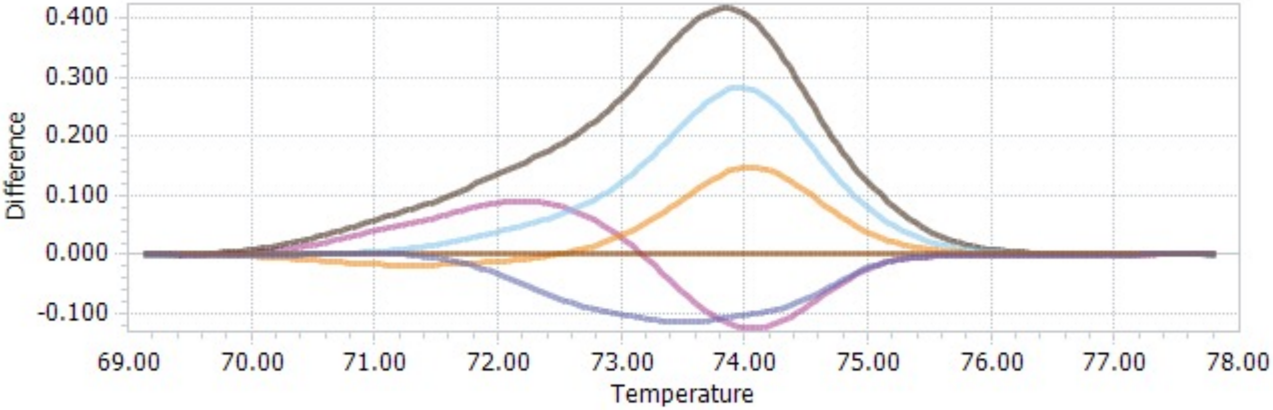
0.400
0.300
0.200
0.100
0.000
-0.100
Difference
69.00
70.00
71.00
72.00
73.00
74.00
75.00
76.00
77.00
78.00
Temperature

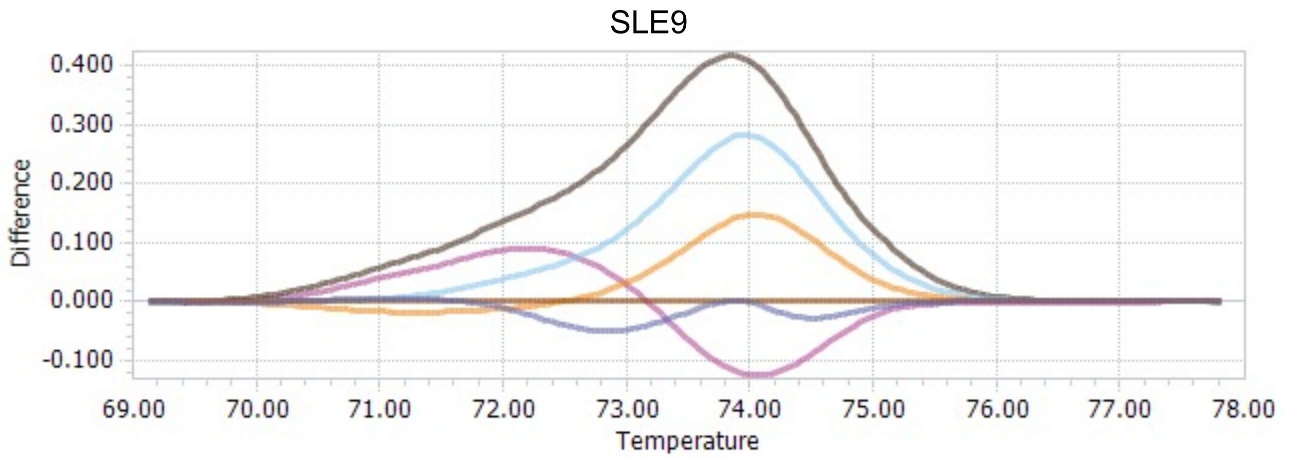

SLE9
0.400
0.300
0.200
0.100
0.000
-0.100
Difference
69.00
70.00
71.00
72.00
73.00
74.00
75.00
76.00
77.00
78.00
Temperature

SLE10

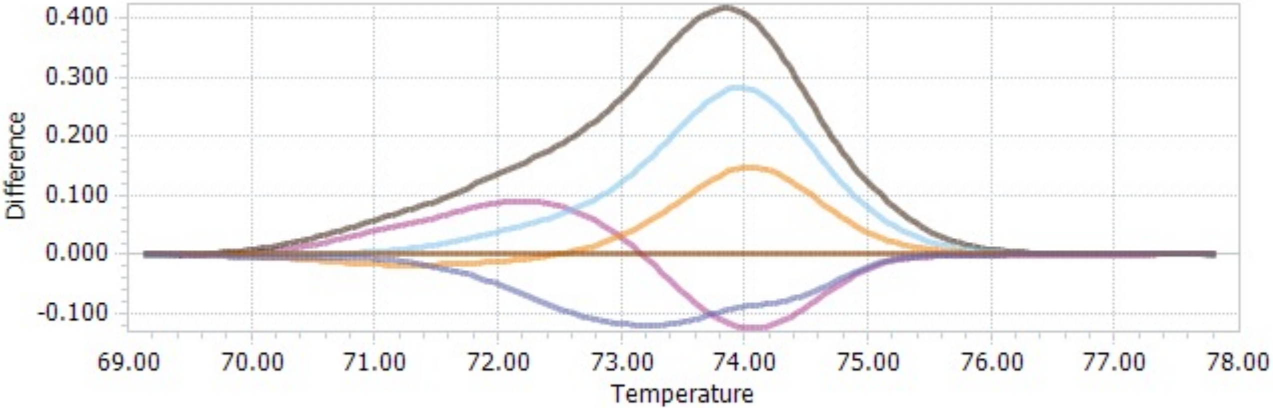

0.400
0.300
0.200
0.100
0.000
-0.100
Difference
69.00
70.00
71.00
72.00
73.00
74.00
75.00
76.00
77.00
78.00
Temperature

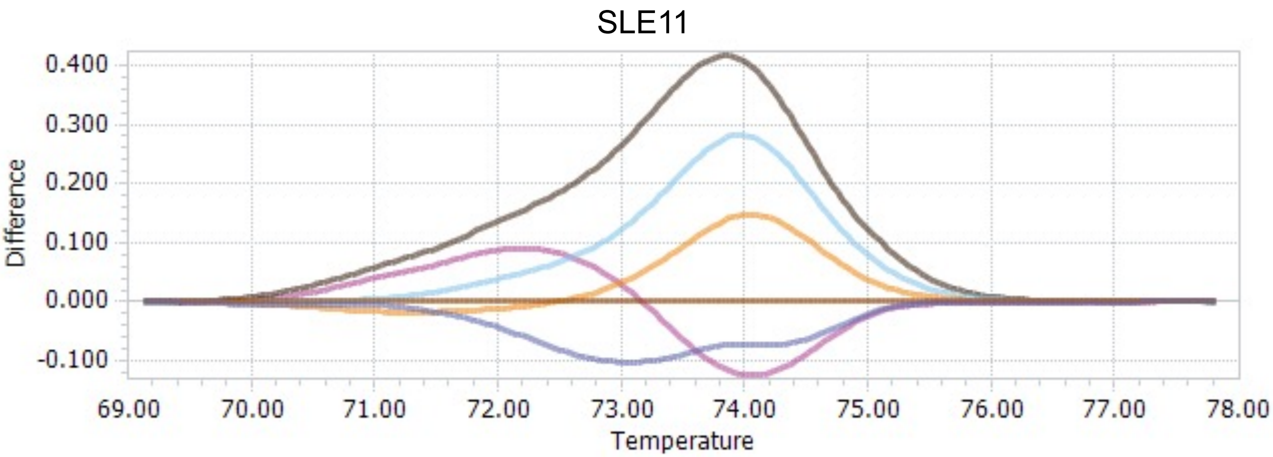
SLE11
Difference
0.400
0.300
0.200
0.100
0.000
-0.100
69.00
70.00
71.00
72.00
73.00
74.00
75.00
76.00
77.00
78.00
Temperature

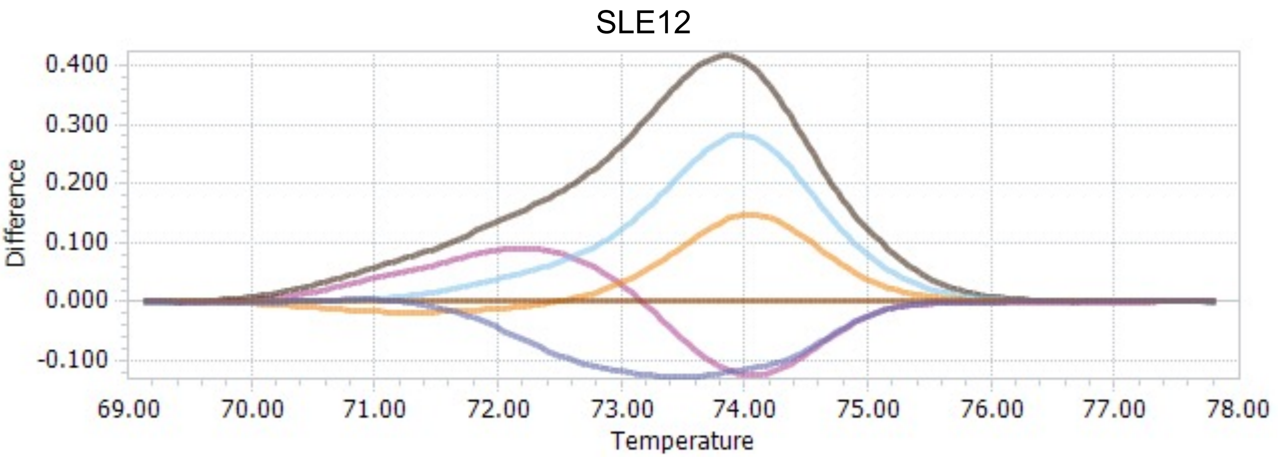
SLE12
Difference
0.400
0.300
0.200
0.100
0.000
-0.100
69.00
70.00
71.00
72.00
73.00
74.00
75.00
76.00
77.00
78.00
Temperature

SLE13

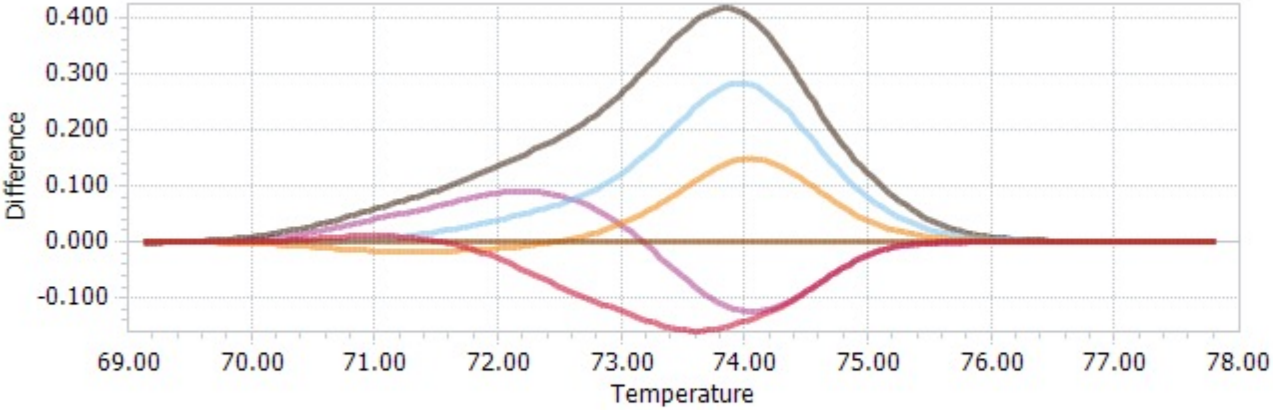
0.400
0.300
0.200
0.100
0.000
-0.100
Difference
69.00
70.00
71.00
72.00
73.00
74.00
75.00
76.00
77.00
78.00
Temperature

SLE14

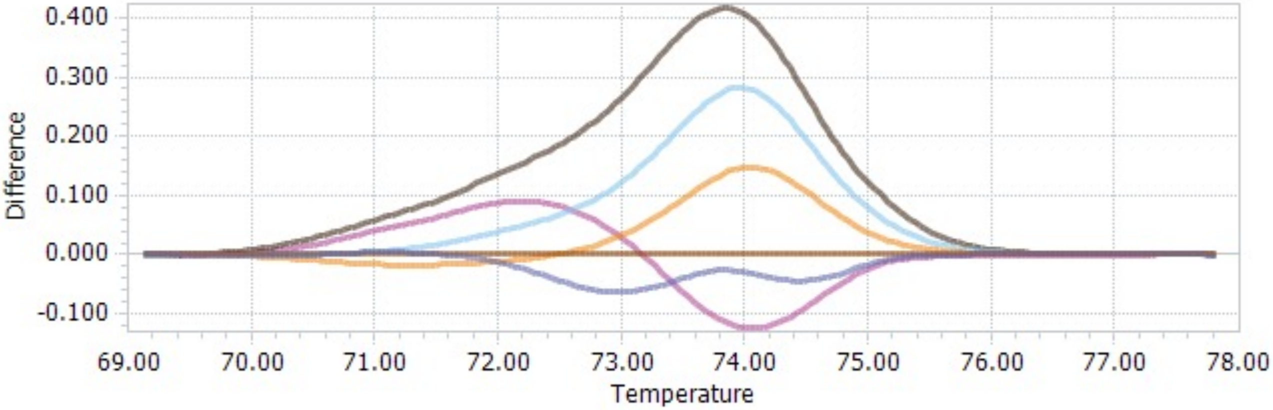

Difference
0.400
0.300
0.200
0.100
0.000
-0.100
69.00
70.00
71.00
72.00
73.00
74.00
75.00
76.00
77.00
78.00
Temperature

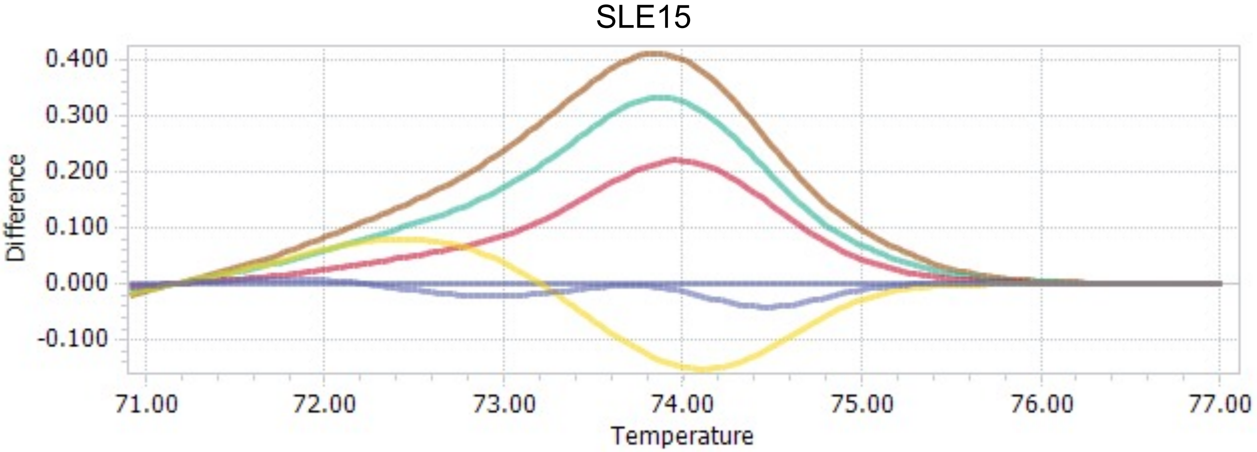
SLE15
Difference
0.400
0.300
0.200
0.100
0.000
-0.100
71.00
72.00
73.00
74.00
75.00
76.00
77.00
Temperature

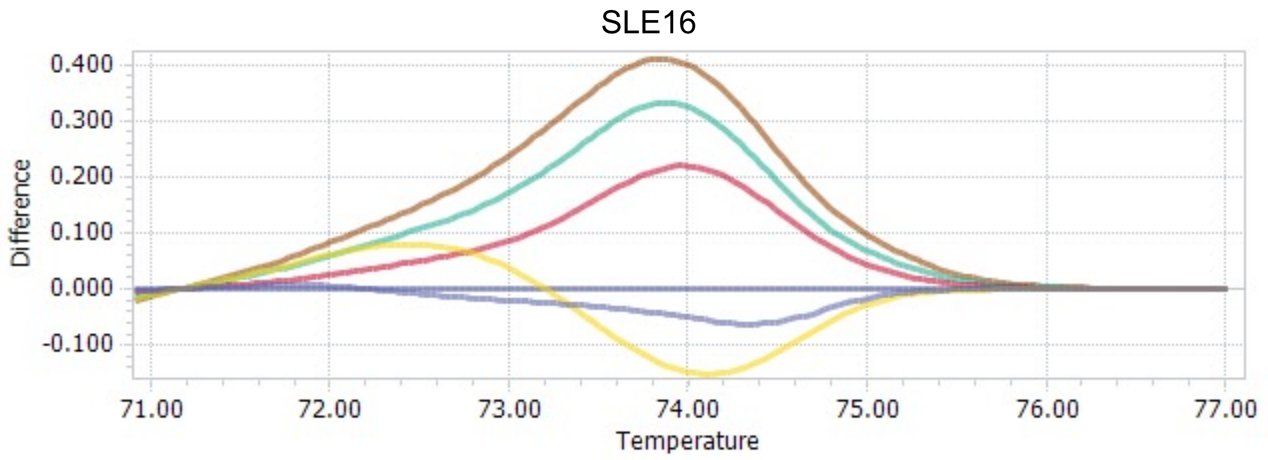
SLE16
Difference
0.400
0.300
0.200
0.100
0.000
-0.100
71.00
72.00
73.00
74.00
75.00
76.00
77.00
Temperature

SLE17

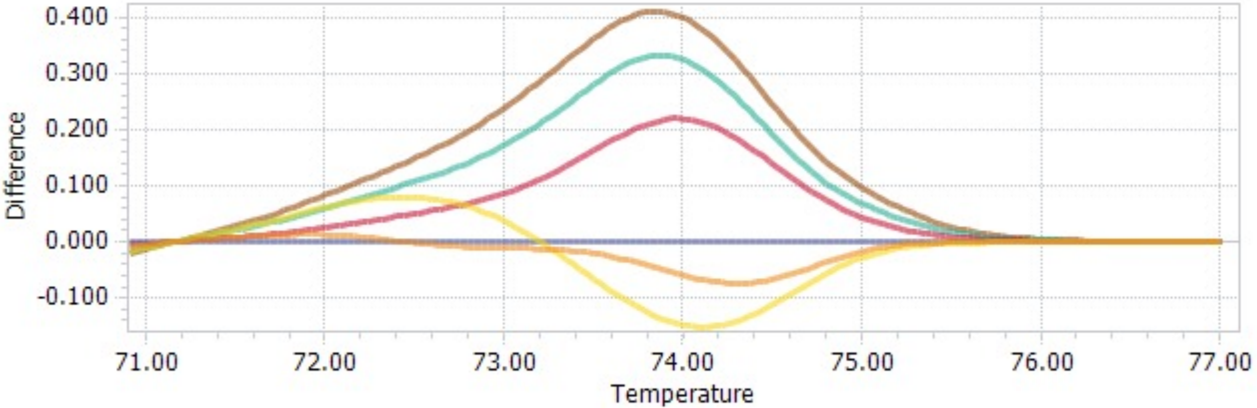

0.400
0.300
0.200
0.100
0.000
-0.100
Difference
71.00
72.00
73.00
74.00
75.00
76.00
77.00
Temperature

SLE18

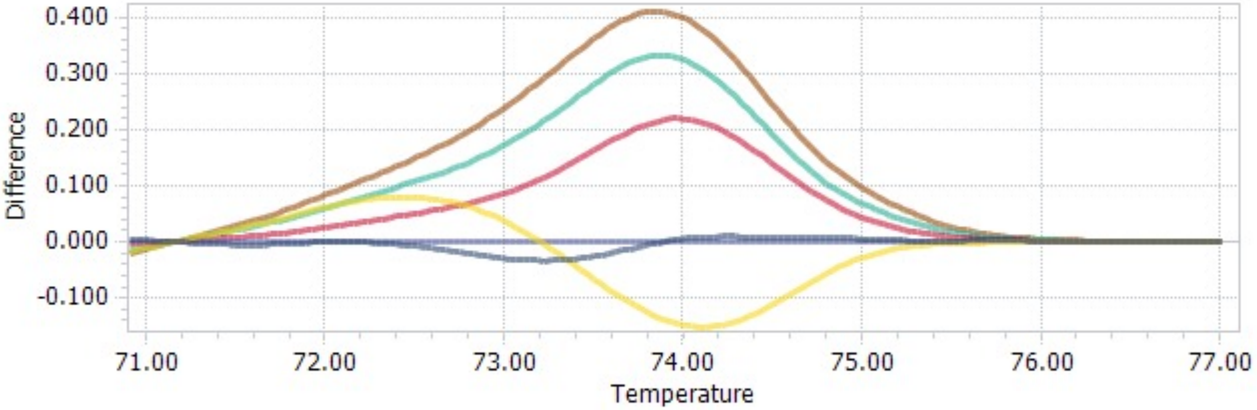
0.400
0.300
0.200
0.100
0.000
-0.100
Difference
71.00
72.00
73.00
74.00
75.00
76.00
77.00
Temperature

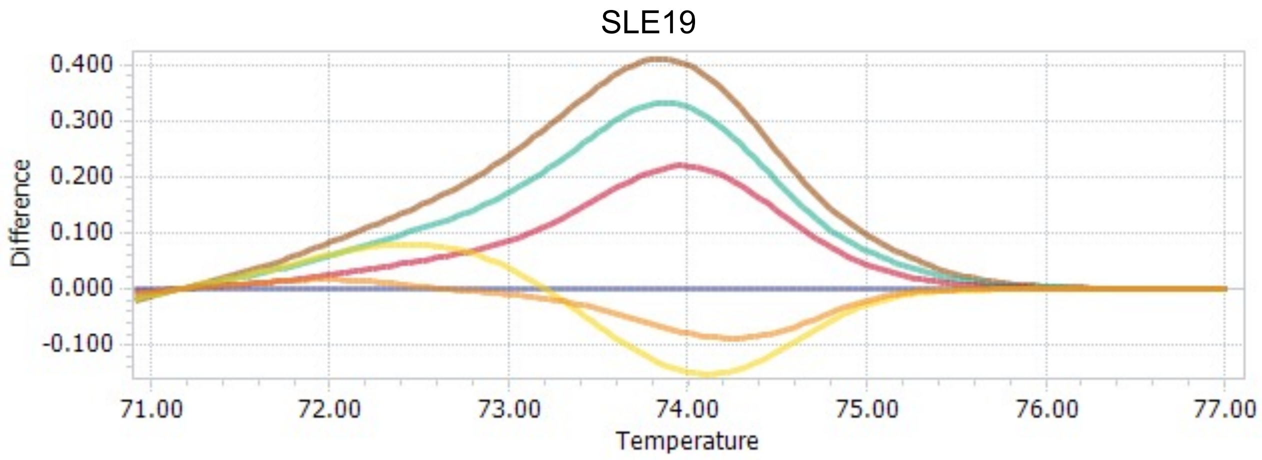
SLE19
0.400
0.300
0.200
0.100
0.000
-0.100
Difference
71.00
72.00
73.00
74.00
75.00
76.00
77.00
Temperature

SLE20

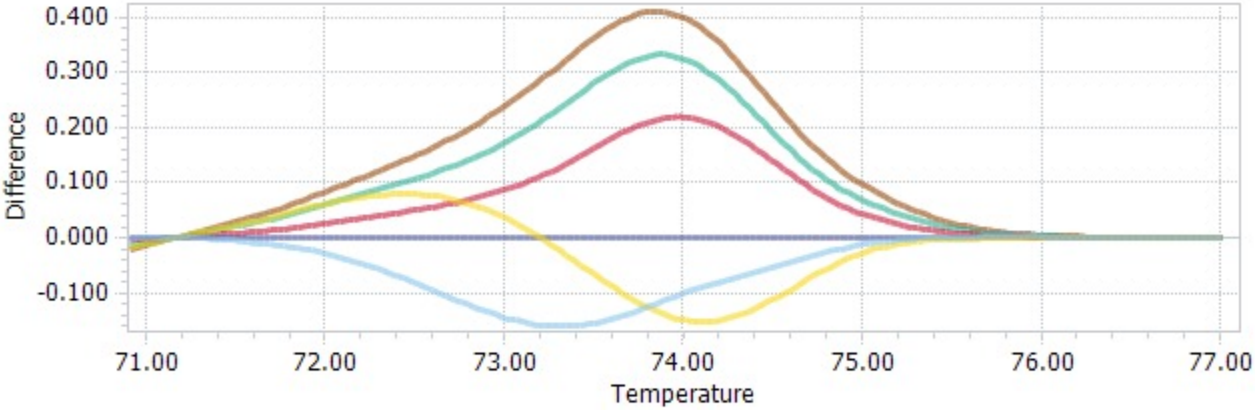
0.400
0.300
0.200
0.100
0.000
-0.100
Difference
71.00
72.00
73.00
74.00
75.00
76.00
77.00
Temperature

SLE21

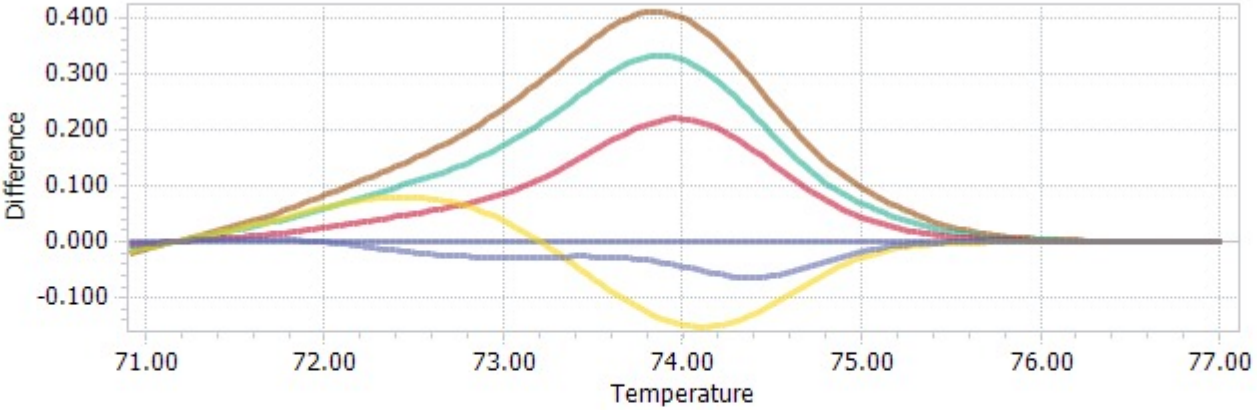
0.400
0.300
0.200
0.100
0.000
-0.100
Difference
71.00
72.00
73.00
74.00
75.00
76.00
77.00
Temperature

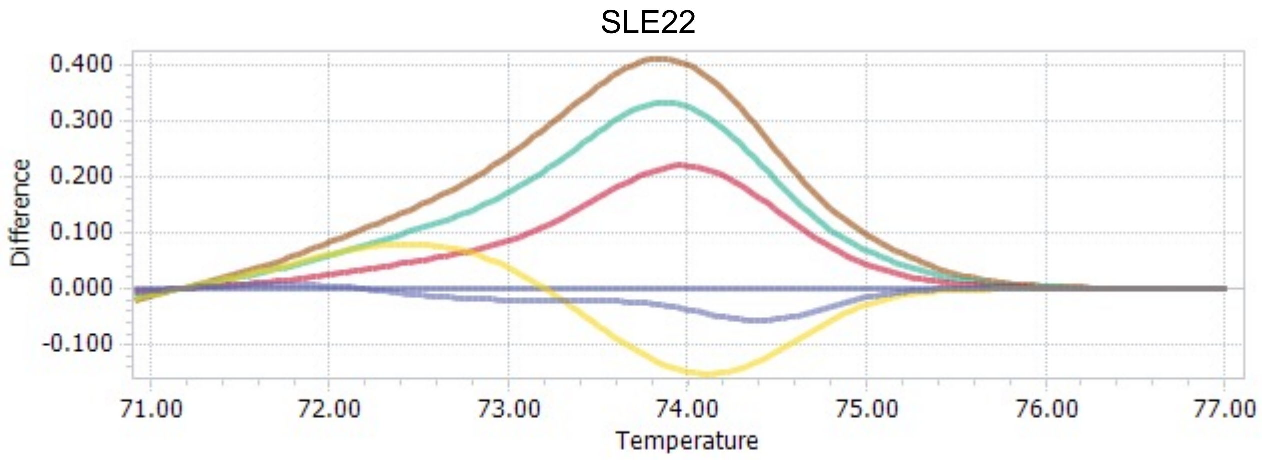
SLE22
Difference
0.400
0.300
0.200
0.100
0.000
-0.100
71.00
72.00
73.00
74.00
75.00
76.00
77.00
Temperature

SLE23

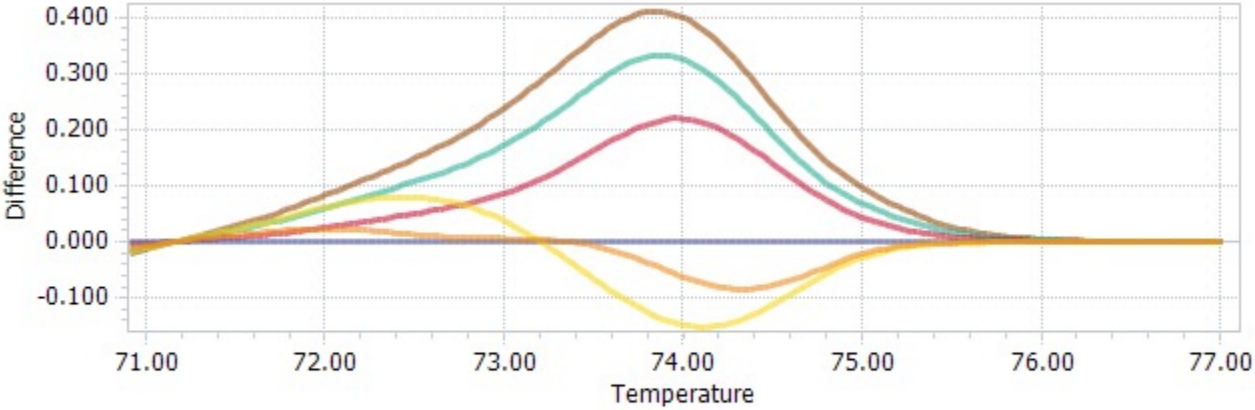
0.400
0.300
0.200
0.100
0.000
-0.100
Difference
71.00
72.00
73.00
74.00
75.00
76.00
77.00
Temperature

SLE24

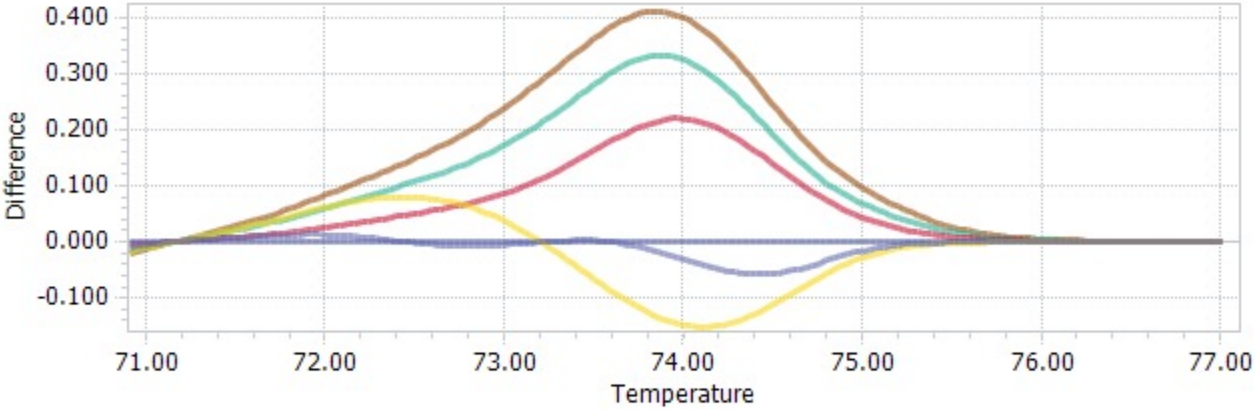

0.400
0.300
0.200
0.100
0.000
-0.100
Difference
71.00
72.00
73.00
74.00
75.00
76.00
77.00
Temperature

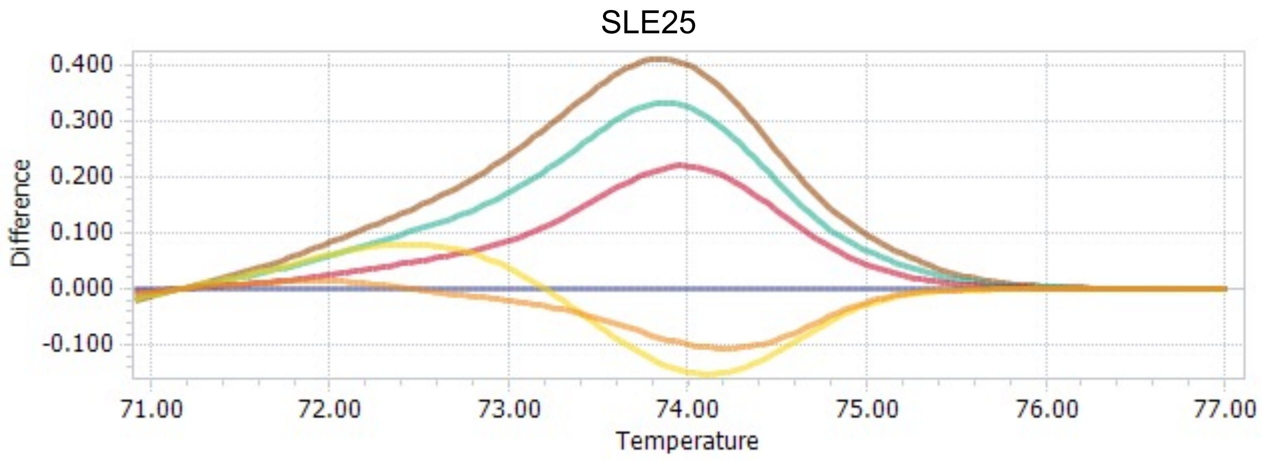
SLE25
Difference
0.400
0.300
0.200
0.100
0.000
-0.100
71.00
72.00
73.00
74.00
75.00
76.00
77.00
Temperature

SLE26

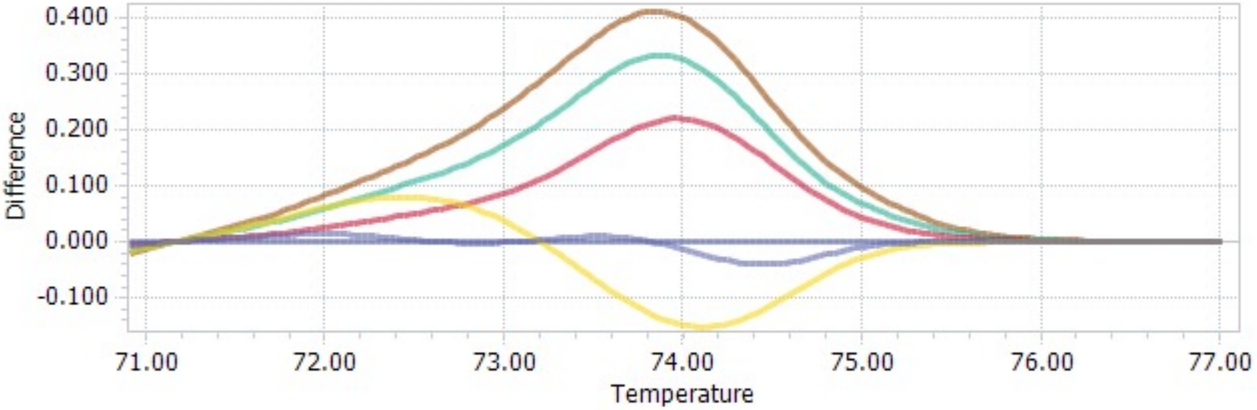
0.400
0.300
0.200
0.100
0.000
-0.100
Difference
71.00
72.00
73.00
74.00
75.00
76.00
77.00
Temperature

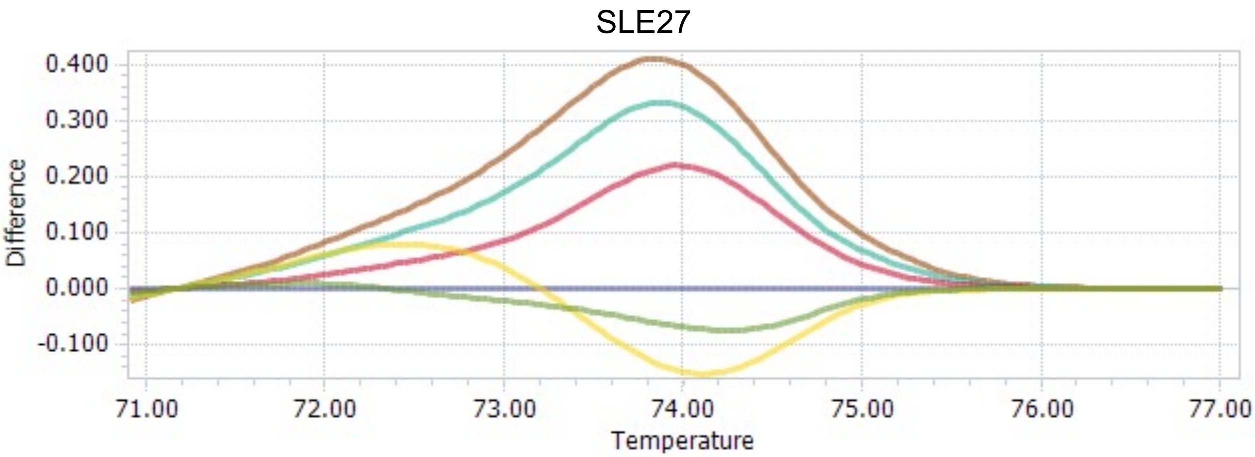
SLE27
0.400
0.300
0.200
0.100
0.000
-0.100
Difference
71.00
72.00
73.00
74.00
75.00
76.00
77.00
Temperature

SLE28

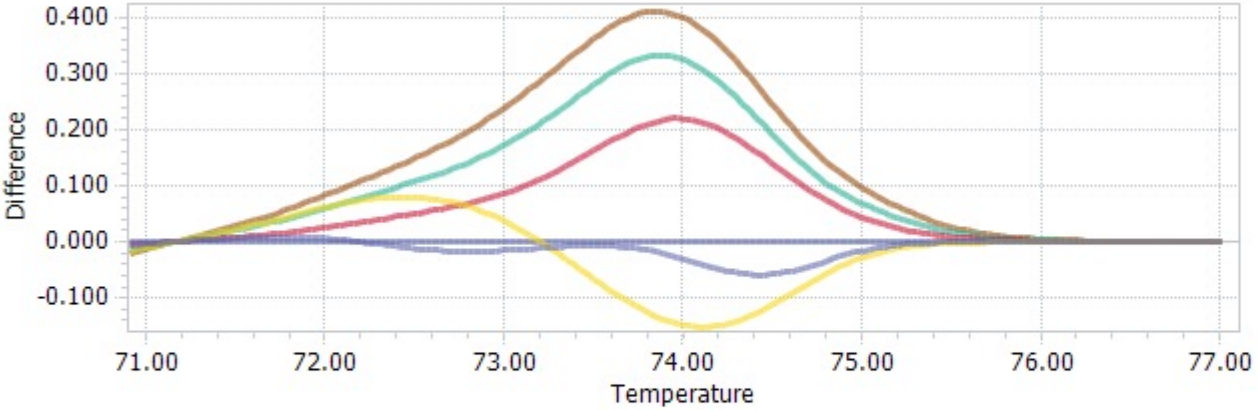

0.400
0.300
0.200
0.100
0.000
-0.100
Difference
71.00
72.00
73.00
74.00
75.00
76.00
77.00
Temperature

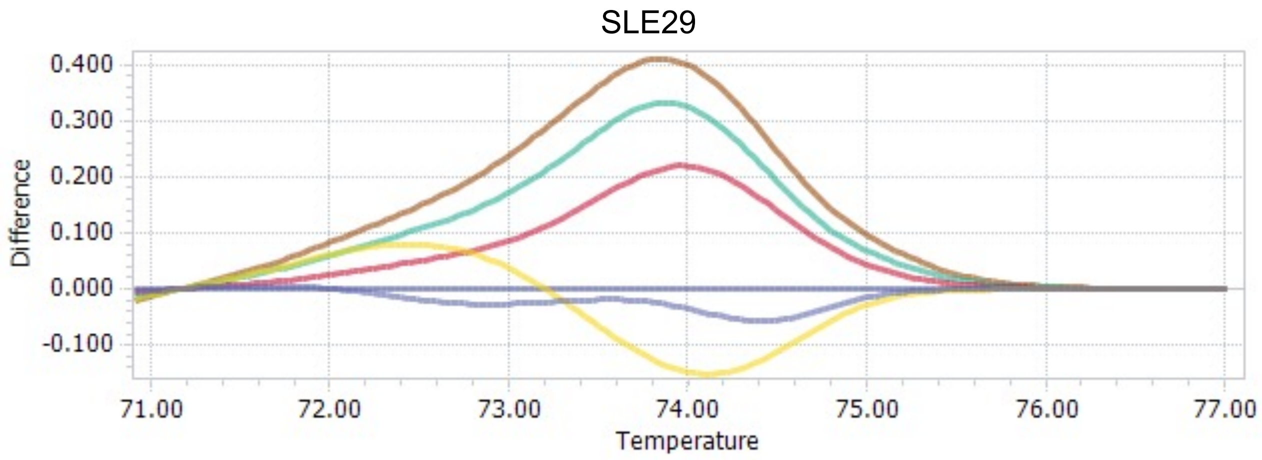
SLE29
Difference
0.400
0.300
0.200
0.100
0.000
-0.100
71.00
72.00
73.00
74.00
75.00
76.00
77.00
Temperature

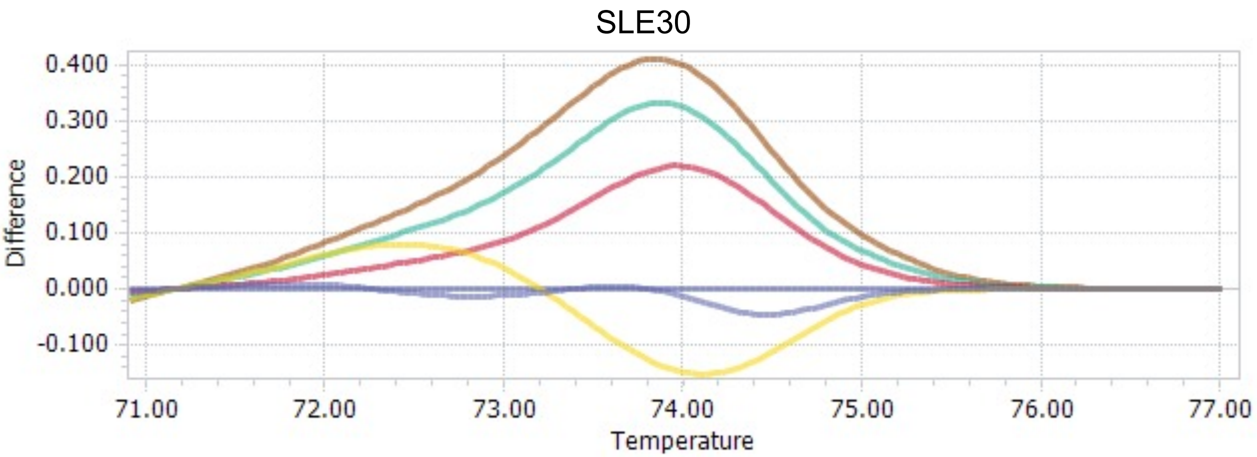
SLE30
Difference
0.400
0.300
0.200
0.100
0.000
-0.100
71.00
72.00
73.00
74.00
75.00
76.00
77.00
Temperature

SLE31

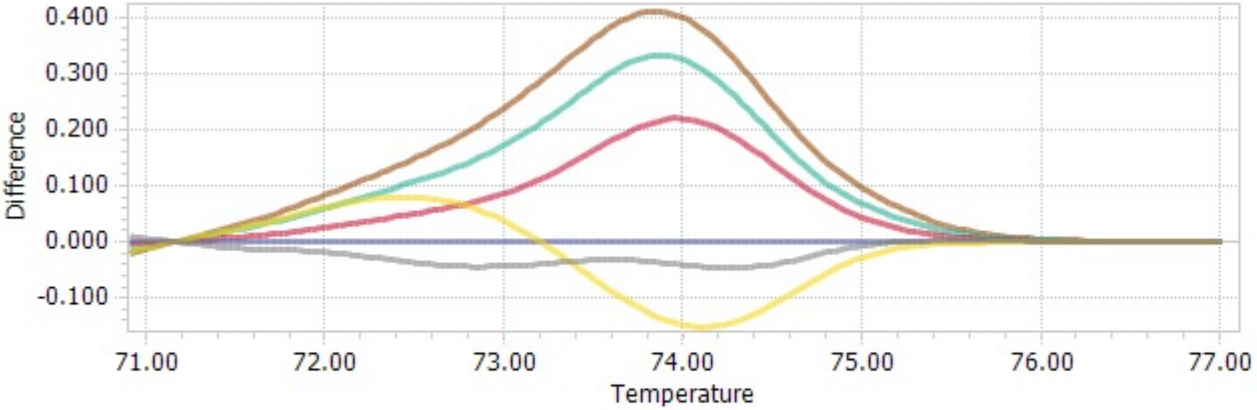

0.400
0.300
0.200
0.100
0.000
-0.100
Difference
71.00
72.00
73.00
74.00
75.00
76.00
77.00
Temperature

SLE32

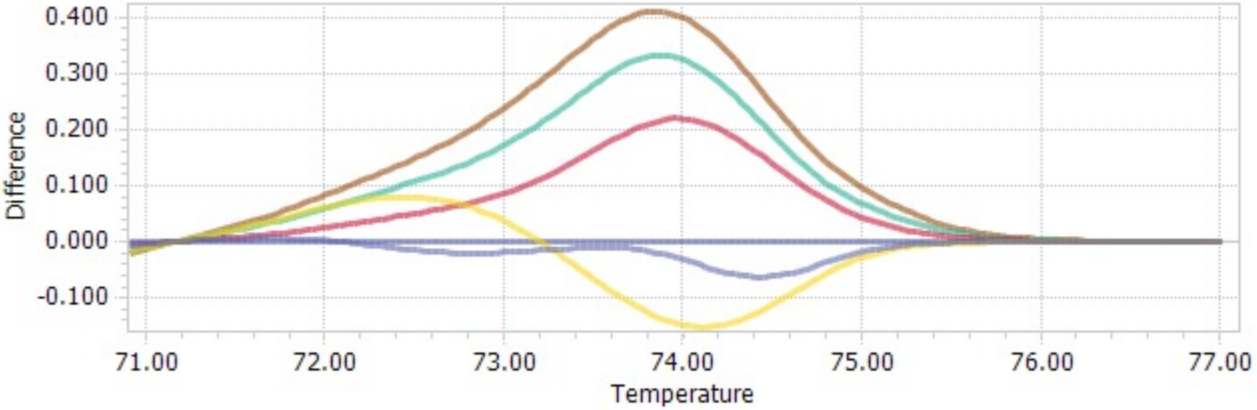
0.400
0.300
0.200
0.100
0.000
-0.100
Difference
71.00
72.00
73.00
74.00
75.00
76.00
77.00
Temperature

SLE33

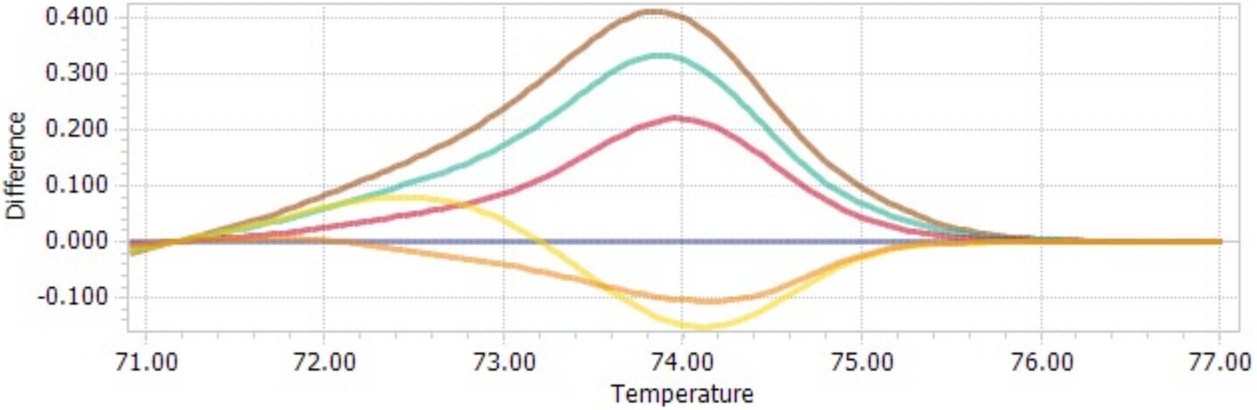

0.400
0.300
0.200
0.100
0.000
-0.100
Difference
71.00
72.00
73.00
74.00
75.00
76.00
77.00
Temperature

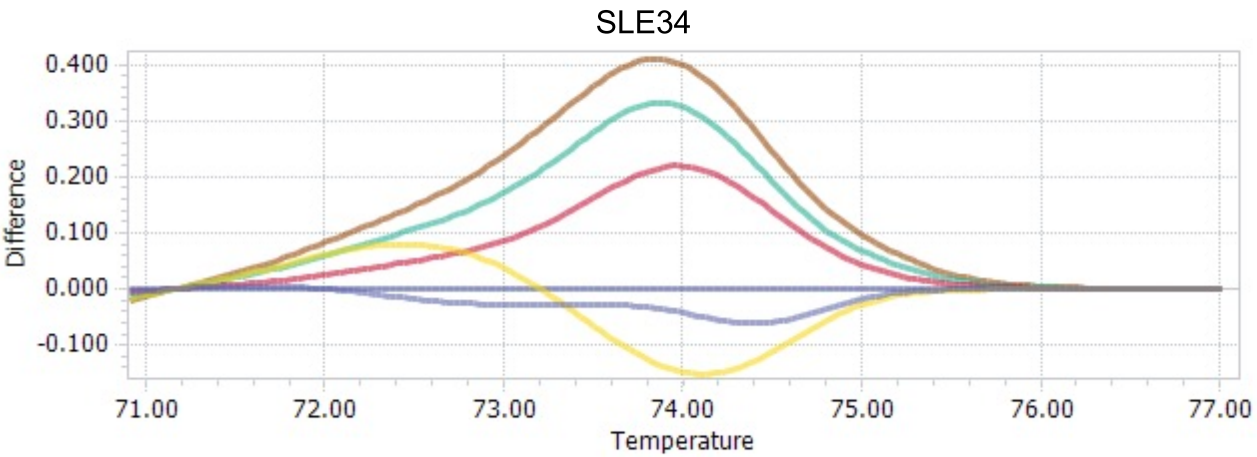

SLE34
Difference
0.400
0.300
0.200
0.100
0.000
-0.100
71.00
72.00
73.00
74.00
75.00
76.00
77.00
Temperature

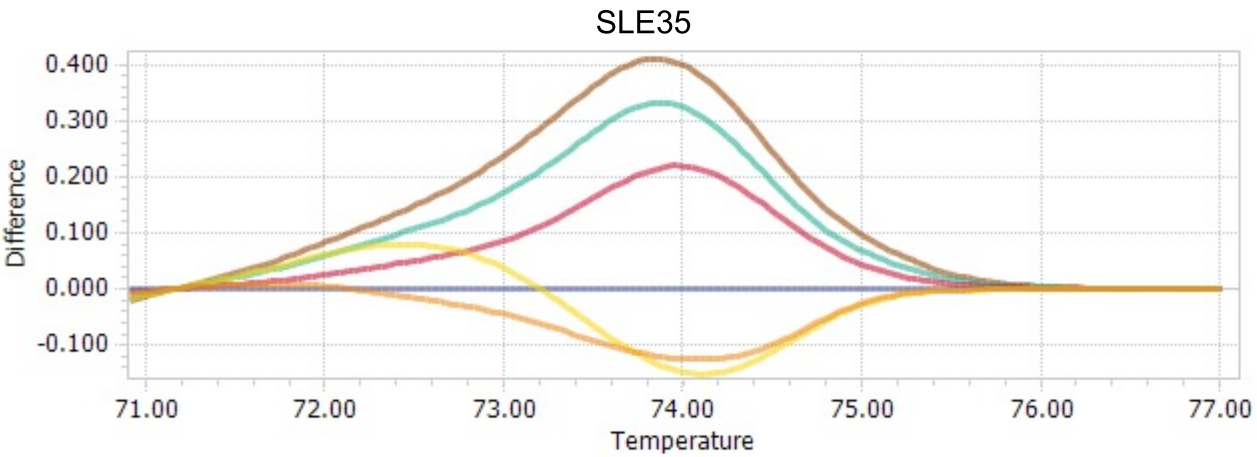

SLE35
0.400
0.300
0.200
0.100
0.000
-0.100
Difference
71.00
72.00
73.00
74.00
75.00
76.00
77.00
Temperature

SLE36

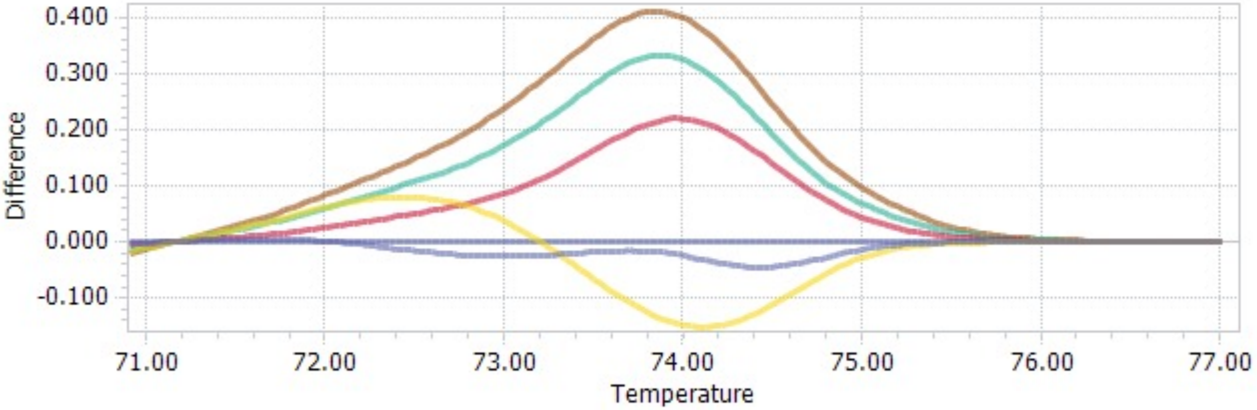

0.400
0.300
0.200
0.100
0.000
-0.100
Difference
71.00
72.00
73.00
74.00
75.00
76.00
77.00
Temperature

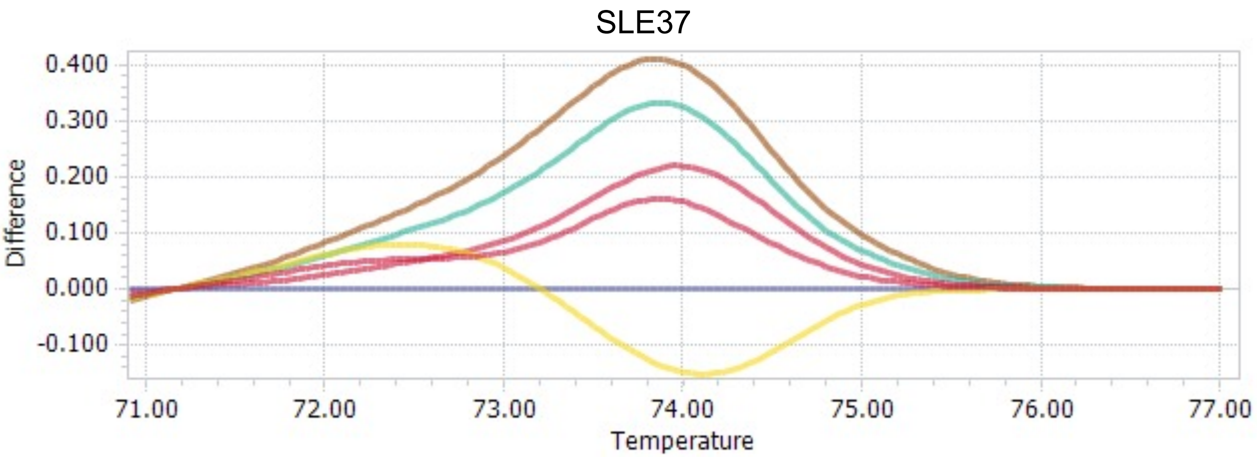
SLE37
Difference
0.400
0.300
0.200
0.100
0.000
-0.100
71.00
72.00
73.00
74.00
75.00
76.00
77.00
Temperature

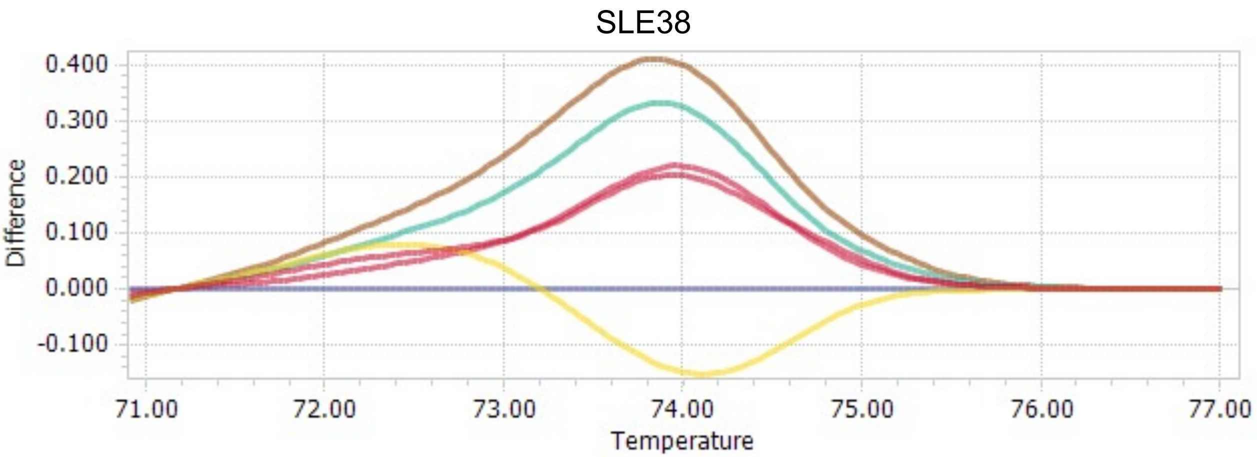
SLE38
Difference
0.400
0.300
0.200
0.100
0.000
-0.100
71.00
72.00
73.00
74.00
75.00
76.00
77.00
Temperature

SLE39

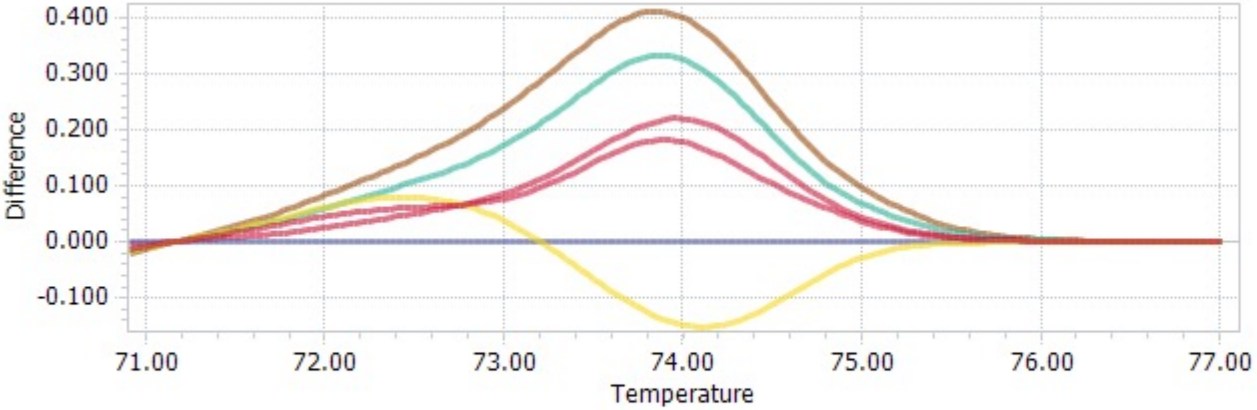

0.400
0.300
0.200
0.100
0.000
-0.100
Difference
71.00
72.00
73.00
74.00
75.00
76.00
77.00
Temperature

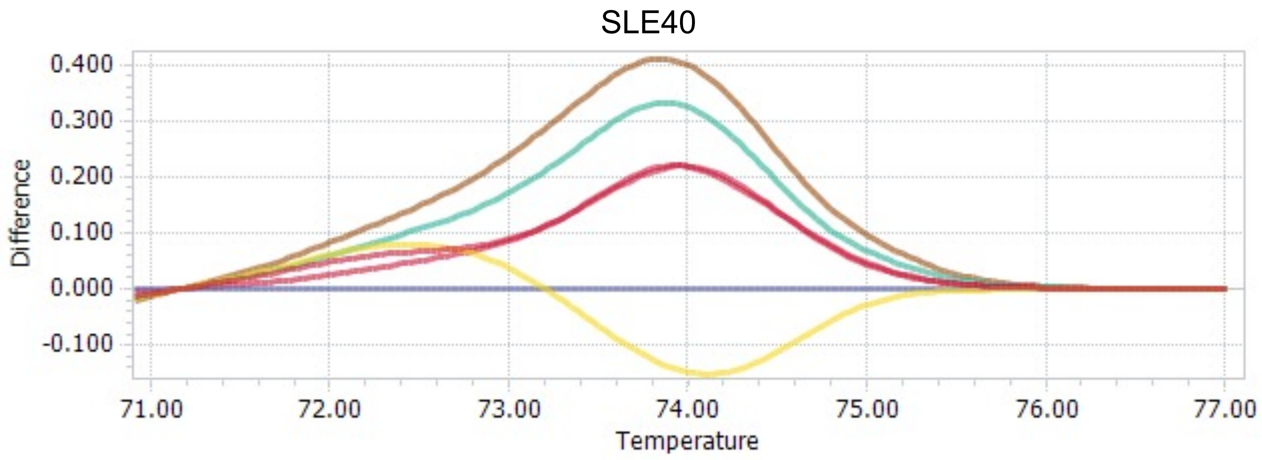
SLE40
Difference
0.400
0.300
0.200
0.100
0.000
-0.100
71.00
72.00
73.00
74.00
75.00
76.00
77.00
Temperature

SLE41

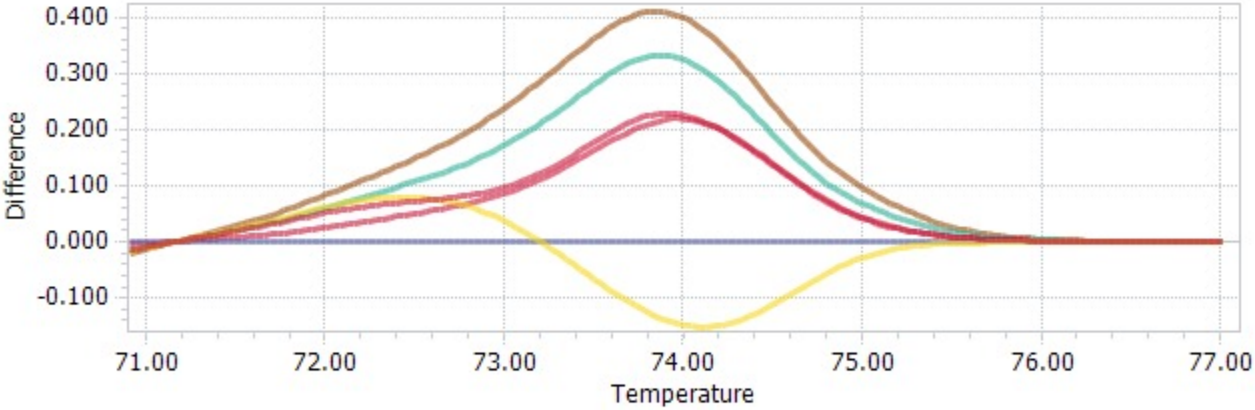

0.400
0.300
0.200
0.100
0.000
-0.100
Difference
71.00
72.00
73.00
74.00
75.00
76.00
77.00
Temperature

SLE42

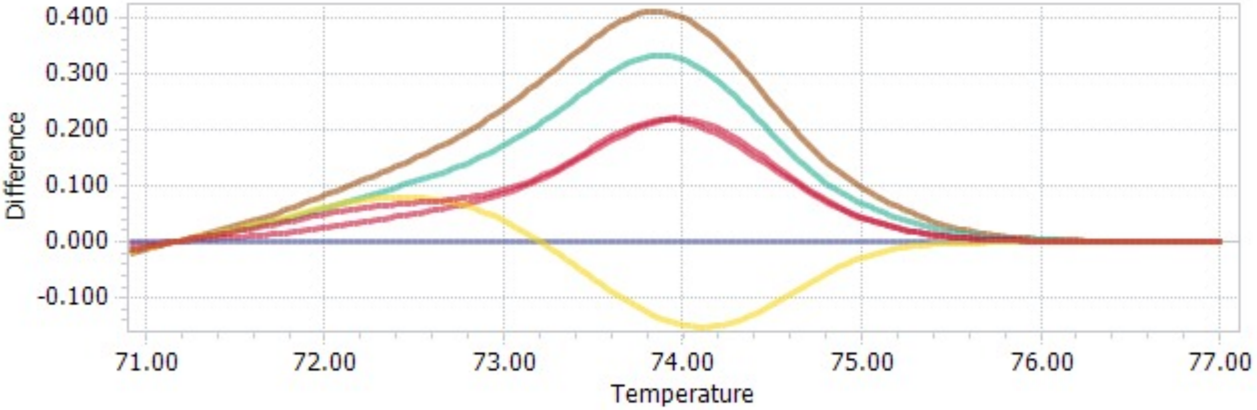
0.400
0.300
0.200
0.100
0.000
-0.100
Difference
71.00
72.00
73.00
74.00
75.00
76.00
77.00
Temperature

SLE43

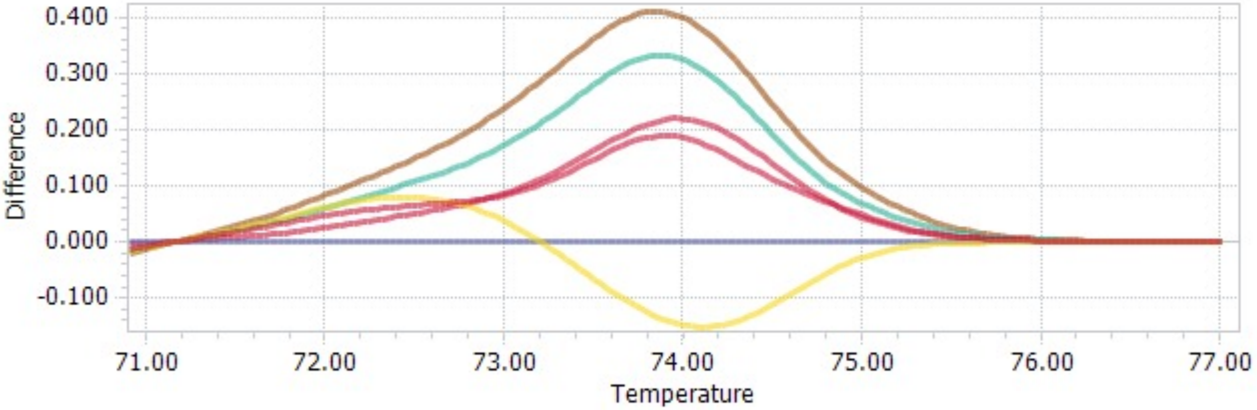
0.400
0.300
0.200
0.100
0.000
-0.100
Difference
71.00
72.00
73.00
74.00
75.00
76.00
77.00
Temperature

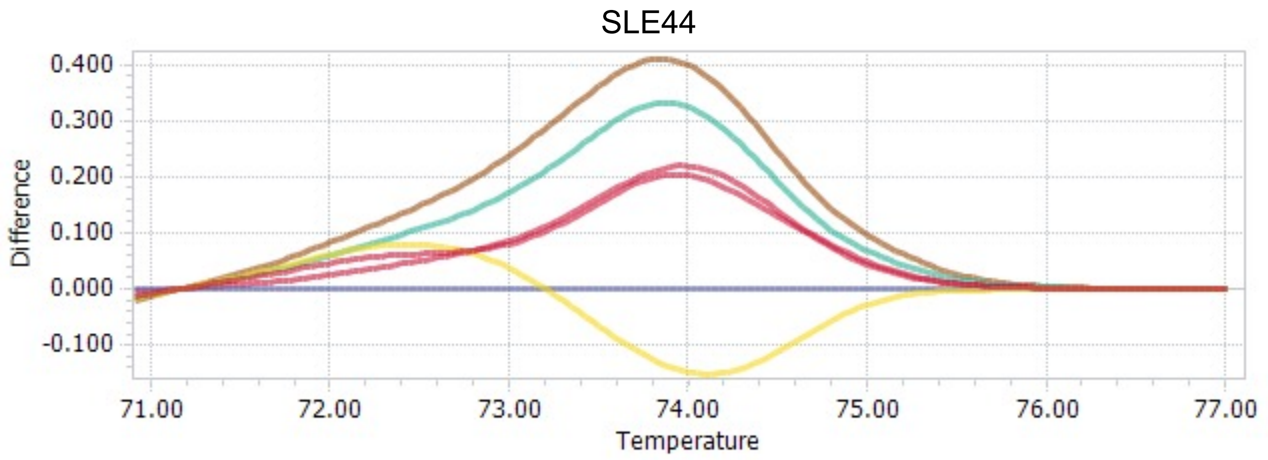
SLE44
0.400
0.300
0.200
0.100
0.000
-0.100
Difference
71.00
72.00
73.00
74.00
75.00
76.00
77.00
Temperature

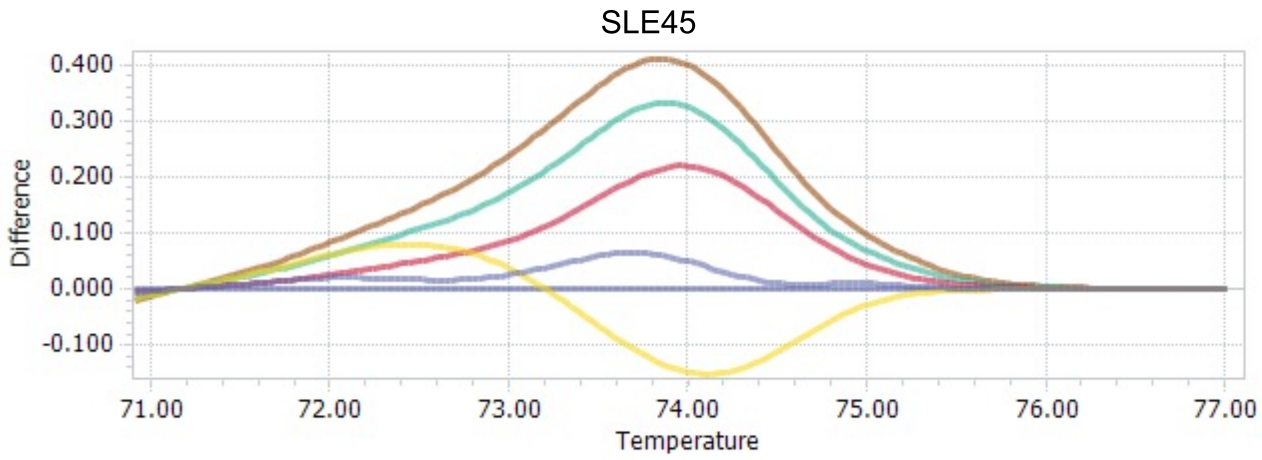
SLE45
0.400
0.300
0.200
0.100
0.000
-0.100
Difference
71.00
72.00
73.00
74.00
75.00
76.00
77.00
Temperature

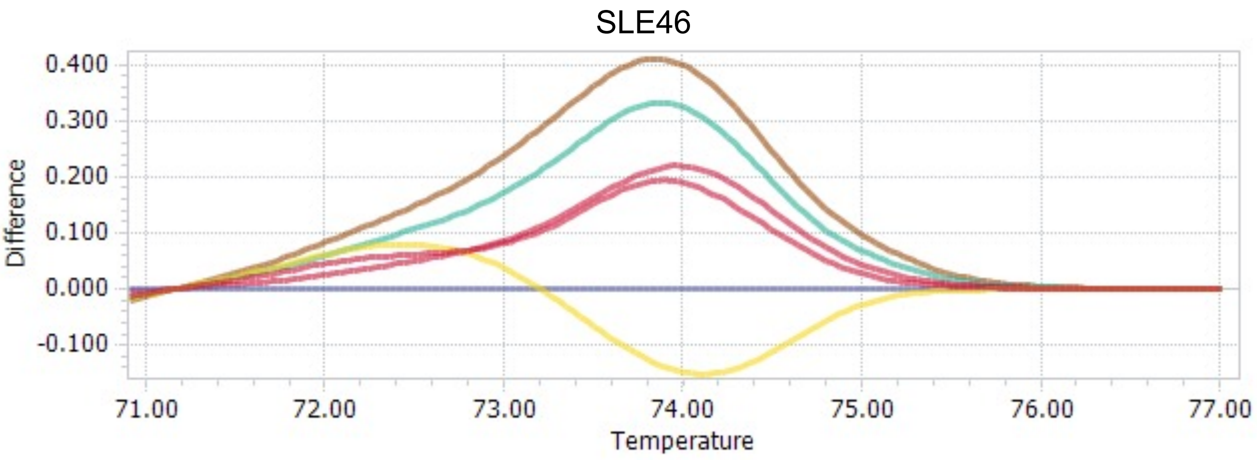
SLE46
Difference
0.400
0.300
0.200
0.100
0.000
-0.100
71.00
72.00
73.00
74.00
75.00
76.00
77.00
Temperature

SLE47

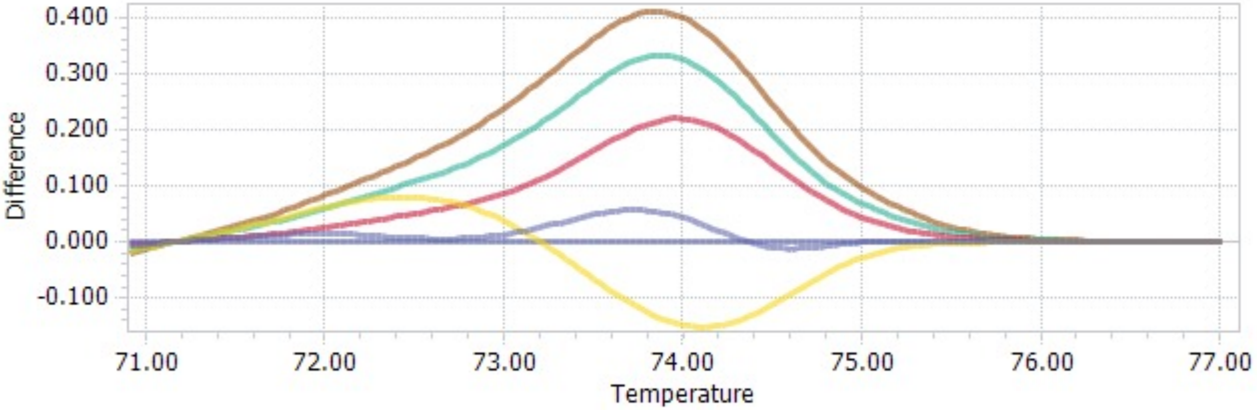

0.400
0.300
0.200
0.100
0.000
-0.100
Difference
71.00
72.00
73.00
74.00
75.00
76.00
77.00
Temperature

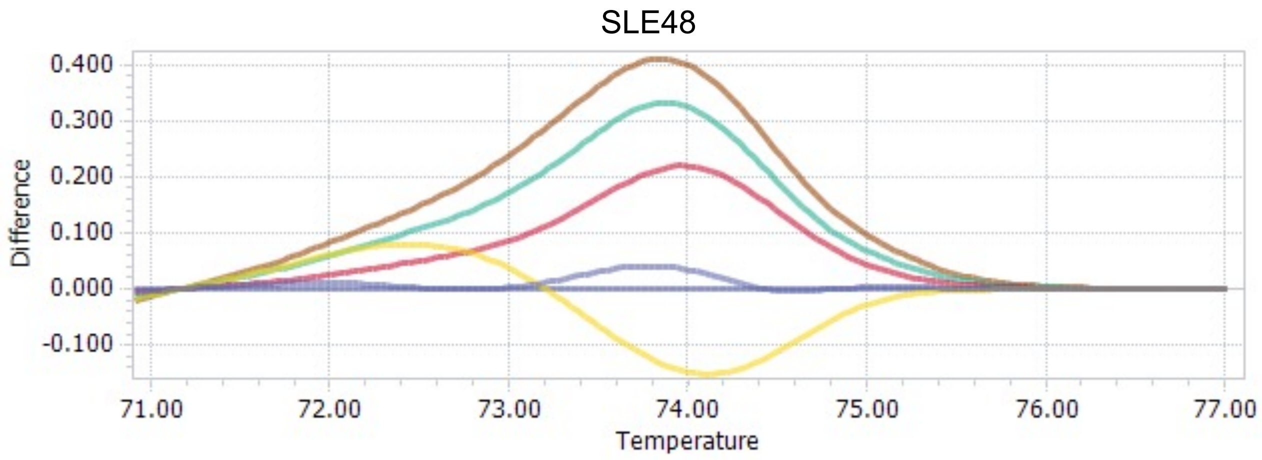
SLE48
0.400
0.300
0.200
0.100
0.000
-0.100
Difference
71.00
72.00
73.00
74.00
75.00
76.00
77.00
Temperature

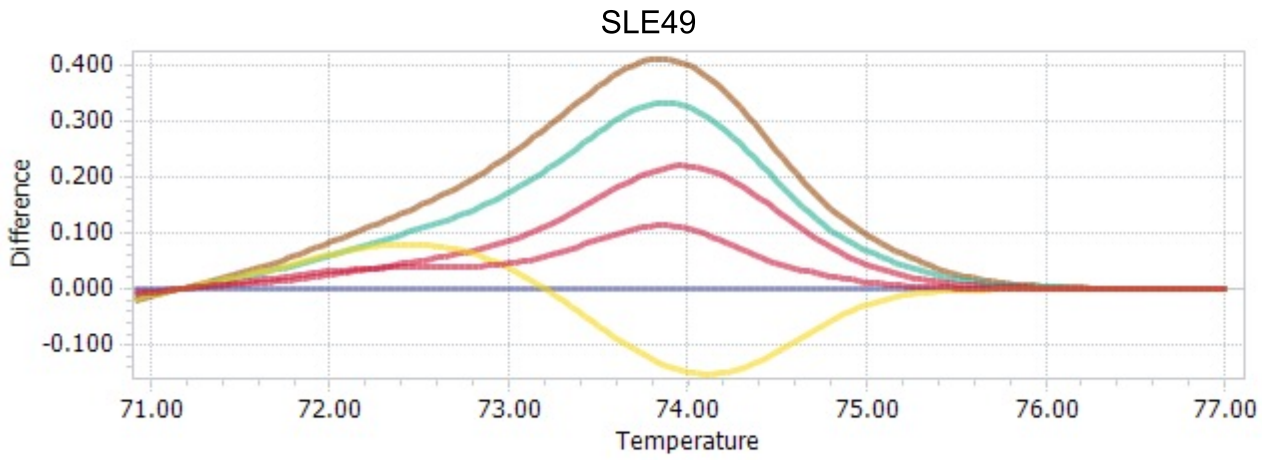
SLE49
0.400
0.300
0.200
0.100
0.000
-0.100
Difference
71.00
72.00
73.00
74.00
75.00
76.00
77.00
Temperature
