# Supplementary material for: DNA methylation of IFI44L as a potential blood biomarker for childhood-onset systemic lupus erythematosus
Source: Pediatr Res. 2024 Mar 21;96(2):494–501. doi: 10.1038/s41390-024-03135-1 (PMC11343705; doi:10.1038/s41390-024-03135-1)
Supplement: Supplementary file 5 — Supplementary 5 [file 41390_2024_3135_MOESM5_ESM.pdf]

中南大学湘雅二医院临床研究伦理委员会
临床研究审查意见

（2022）伦审【临研】第（063）号

<table>
<tr><td>项目名称</td><td colspan="5">儿童 SLE 精准诊疗体系的研究</td></tr>
<tr><td>发起单位</td><td colspan="5">中南大学湘雅二医院</td></tr>
<tr><td>主要研究者</td><td colspan="2">吴小川/主任医师</td><td>专业</td><td colspan="2">儿科</td></tr>
<tr><td>研究性质</td><td colspan="5">☑ 回顾性　☐ 诊断性　☑ 干预性　☑ 生物标本利用
☐ 预后观察性　☐ 问卷调查　☐ 其他</td></tr>
<tr><td>经费来源</td><td colspan="5">中央财政专项资金</td></tr>
<tr><td>审查方式</td><td colspan="2">■会议审查　☐快速审查</td><td>审查地点</td><td colspan="2">精卫楼 19 楼机构会议室</td></tr>
<tr><td>委员人数</td><td>13 人</td><td>到会委员人数</td><td>13 人</td><td>回避委员</td><td>无</td></tr>
<tr><td>邀请专家</td><td>无</td><td>材料形式审查</td><td colspan="3">■合格　☐不合格</td></tr>
<tr><td>审阅及审评文件</td><td colspan="5">1. 临床试验方案（方案编号:2021YFC2702004、版本号:1.0、版本日期:2021.12.16);
2. 知情同意申请书（版本号：1.0、版本日期：2021.12.16);
3. 研究者简历;
4. 参加中心列表;
5. 病例报告表(版本号：1.0、版本日期：2021.12.16);
6. 风险处置预案(版本号：1.0、版本日期：2021.12.16);
7. 人类遗传资源管理承诺书;</td></tr>
<tr><td>伦理委员会意见</td><td colspan="5">■同意　☐作必要修改后同意　☐不同意　☐终止或暂停已批准的试验
备注：</td></tr>
<tr><td>跟踪审查频率</td><td colspan="5">☐3 个月　☐6 个月　■12 个月　☐不适用</td></tr>
<tr><td>批件有效期</td><td colspan="2">一年</td><td>批件失效日期</td><td colspan="2">2023 年 3 月 29 日</td></tr>
<tr><td colspan="6">主任委员/授权副主任委员签名：[signature]　日期：2022.3.30
中南大学湘雅二医院临床研究伦理委员会（盖章）</td></tr>
</table>

中南大学湘雅二医院临床研究伦理委员会
伦理审评会签到表

会议时间：2022 年 3 月 30 日　　　　会议地点：精卫楼 19 楼机构会议室

姓名	性别	职称	工作单位	签名
陈晋东（主任委员）	男	主任医师 教授	中南大学湘雅二医院	
唐罗生（副主任委员）	男	主任医师 教授	中南大学湘雅二医院	
罗爱静（委员）	女	主任医师 教授	中南大学湘雅二医院	
黄江生（委员）	男	主任医师 教授	中南大学湘雅二医院	
陶澄（委员）	男	主任医师 教授	中南大学湘雅二医院	
李霞（委员）	女	主任医师 教授	中南大学湘雅二医院	
吴尚洁（委员）	女	主任医师 教授	中南大学湘雅二医院	
张湘瑜（委员）	女	主任医师 教授	中南大学湘雅二医院	
张毕奎（委员）	男	主任药师 教授	中南大学湘雅二医院	
吴忠仕（委员）	男	主任医师 教授	中南大学湘雅二医院	
李卓（委员）	女	副主任医师 副教授	中南大学湘雅二医院	
任国峰（委员）	男	教授	中南大学公共卫生学院	
罗冰（委员）	女	律师	湖南弘湘律师事务所	

保密协议及利益冲突声明：作为中南大学湘雅二医院临床研究伦理委员会成员，我将对我所审阅的临床研究资料（详见审评文件目录）以及临床研究伦理委员会会议讨论的结果和相关内容保密，并保证所审查的项目与本临床研究伦理委员会成员无任何利益冲突。
